# Nutrient Dense, Low-Cost Foods Can Improve the Affordability and Quality of the New Zealand Diet—A Substitution Modeling Study

**DOI:** 10.3390/ijerph18157950

**Published:** 2021-07-27

**Authors:** Carlene S. Starck, Michelle Blumfield, Tim Keighley, Skye Marshall, Peter Petocz, Elif Inan-Eroglu, Kylie Abbott, Tim Cassettari, Ajmol Ali, Carol Wham, Rozanne Kruger, Geoff Kira, Flavia Fayet-Moore

**Affiliations:** 1 Department of Translational Science, Nutrition Research Australia, Sydney, NSW 2000, Australia; carlene@nraus.com (C.S.S.); tim@nraus.com (T.C.); 2 Riddet Institute, Massey University, Palmerston North 4474, New Zealand; 3 Department of Science, Nutrition Research Australia, Sydney, NSW 2000, Australia; michelle@nraus.com (M.B.); tim.keighley@gmail.com (T.K.); skye@nraus.com (S.M.); peter.petocz@mq.edu.au (P.P.); elif.inaneroglu@sydney.edu.au (E.I.-E.); 4 Bond University Nutrition & Dietetics Research Group, Faculty of Health Sciences and Medicine, Bond University, Gold Coast, QLD 4226, Australia; 5 Charles Perkins Centre, School of Health Sciences, Faculty of Medicine and Health, The University of Sydney, Camperdown, NSW 2000, Australia; 6 Nutrition Research Australia, Sydney, NSW 2000, Australia; kylie@nraus.com; 7 School of Sport, Exercise and Nutrition, College of Health, Massey University, North Shore City, Auckland 0745, New Zealand; A.Ali@massey.ac.nz (A.A.); C.A.Wham@massey.ac.nz (C.W.); R.Kruger@massey.ac.nz (R.K.); 8 School of Health Sciences, College of Health, Massey University, Palmerston North 4474, New Zealand; G.Kira@massey.ac.nz

**Keywords:** diet, cost and cost analysis, food security, food quality, socioeconomic factors, ethnic groups, nutrients, economic models

## Abstract

The high prevalence of non-communicable disease in New Zealand (NZ) is driven in part by unhealthy diet selections, with food costs contributing to an increased risk for vulnerable population groups. This study aimed to: (i) identify the nutrient density-to-cost ratio of NZ foods; (ii) model the impact of substituting foods with a lower nutrient density-to-cost ratio with those with a higher nutrient density-to-cost ratio on diet quality and affordability in representative NZ population samples for low and medium socioeconomic status (SES) households by ethnicity; and (iii) evaluate food processing level. Foods were categorized, coded for processing level and discretionary status, analyzed for nutrient density and cost, and ranked by nutrient density-to-cost ratio. The top quartile of nutrient dense, low-cost foods were 56% unprocessed (vegetables, fruit, porridge, pasta, rice, nuts/seeds), 31% ultra-processed (vegetable dishes, fortified bread, breakfast cereals unfortified <15 g sugars/100 g and fortified 15–30 g sugars/100 g), 6% processed (fruit juice), and 6% culinary processed (oils). Using substitution modeling, diet quality improved by 59% and 71% for adults and children, respectively, and affordability increased by 20–24%, depending on ethnicity and SES. The NZ diet can be made healthier and more affordable when nutritious, low-cost foods are selected. Processing levels in the healthier, modeled diet suggest that some non-discretionary ultra-processed foods may provide a valuable source of low-cost nutrition for food insecure populations.

## 1. Introduction

Ultra-processed and discretionary foods (non-core foods that do not contribute to a healthy diet) [1,2] are overrepresented in the New Zealand (NZ) diet; contributing to high intakes of saturated fat, salt, and sugars, but low intakes of essential nutrients such as calcium, zinc, vitamin A, and selenium [1,3,4]. These unhealthy dietary patterns have contributed to an increased incidence of non-communicable diseases (NCDs) in NZ [5] (e.g., cardiovascular diseases, cancer, and diabetes), with NCDs being the country’s leading cause (89%) of adult death [6]. Inequities in the prevalence of NCDs and their potentially modifiable risk factors exist within different groups [6,7], with differences most pronounced for Māori, Pacific peoples, and people living in socio-economically disadvantaged areas [7]. The amenable mortality rate is 2.5 times higher for Māori and Pacific peoples than for NZ European and other ethnic groups (NZEO) [7]; and those living in low socio-economic status (SES) areas have higher prevalence of NCD risk factors, compared high SES areas [8]. Improving the quality of the NZ diet across all ethnic and socioeconomic populations is of critical public health importance [9].

The food environment, particularly food cost, is a major factor preventing access to, and consumption of, healthy diets [3]. Dietary modeling in NZ suggests that a healthy diet is unaffordable for low SES families as it can require up to 50% of household income [1], which often leads to families selecting low-cost foods that are energy dense, nutrient poor, and ultra-processed [4]. Māori and Pacific populations are disproportionately represented in lower SES areas, and thus more likely to experience food insecurity (adults were 16% Māori and 21% Pacific) compared to NZEO (6% of adults) [6], particularly across the SES indictors of education, employment, income, and household crowding [7]. Differences are further highlighted by a lower intake of fruit and vegetables in Pacific adults [8,10] and a higher intake of processed meat, margarine, confectionary, and alcohol in Māori adults, compared to other ethnicities [10]. According to the 2019/20 NZ Health Survey, 9.9% of Māori and 20% of Pacific children aged 2–14 years consumed fast food three or more times per week, compared to 3.6% of European-descent children [11]. Further, 52.7% of European-descent children achieved the recommended daily intake of vegetables, compared to 42.2% and 27.7% of Māori and Pacific children, respectively [11]. Ethnic discrepancies are also evident in the intake of fruit, soft drinks, and consumption of breakfast at home [12]. The health and economic pressures caused by the COVID-19 pandemic have placed additional stress on both nutrition and food costs [9]. Therefore, an understanding of NZ foods that are nutritious and low in cost is urgently required, with a focus on culturally-relevant foods and SES [13]. 

Investigations in the USA and Australia have identified the following foods as nutrient dense and low-cost: milk, fruit juice, potatoes, and breakfast cereals [14,15]. In NZ, diets that are considered healthy, low-cost, and environmentally sustainable include carrots, wholemeal flour, pasta, milk powder, and eggs [16]. When food categories in the Australian diet were substituted with those that had a higher nutrient density-to-cost ratio, the quality and affordability of the Australian diet improved, and included approximately 25% ultra-processed foods [15]. There is some evidence that a healthy, low-cost diet is possible in NZ for all ethnic groups [1,17]; however, when the cost of the current versus healthy diets were matched for energy intake, the healthy diet was more expensive, and neither processing level nor the impact of SES on diet affordability was examined [1,17]. It is unclear which NZ foods provide the highest nutrient density for the lowest cost, whether these foods are ultra-processed, and how these foods affect diet quality and affordability for different SES households and ethnic populations. 

Therefore to meet these research gaps, this study aims to: (i) identify the nutrient density-to-cost ratio of NZ foods, with the inclusion of foods frequently consumed by Māori and Pacific population groups; (ii) model the impact of substituting foods with a lower nutrient density-to-cost ratio with those with a higher nutrient density-to-cost ratio on diet quality and affordability in representative NZ population samples for low and medium SES households by ethnicity; and (iii) evaluate food processing level across diets. 

## 2. Materials and Methods

This dietary and economic modeling study was carried out according to the INFORMAS protocol [18], using cross-sectional data. The INFORMAS protocol provides methodology to systematically collect and analyze information on the price of foods, meals, and affordability of diets [18]. The study was reported according to the Nga Tikanga Paihere framework [19] and the Consolidated Health Economic Evaluation Reporting Standards (CHEERS) Statement [20]. 

### 2.1. Nutrient and Food Price Databases

#### 2.1.1. NZ Food and Nutrient Database and Selection of Representative Foods

The NZ Food Composition Database (NZFCDB) [21] contains macro- and micronutrient composition data for over 2700 NZ foods, and is the primary source of food composition data for estimating nutrient intake in NZ nutrition surveys. The NZFCDB organizes individual foods into 23 major food chapters, each coded by a single letter. Individual foods within each chapter are given a number, to create an alpha-numerical identifier for each food.

In the current study, NZFCDB foods were aggregated into food categories based on the major food chapters and modification of a previously published aggregation of the AUSNUT 2011–2013 nutrient database, to allow for both sufficient complexity and sensitivity in the modeling [15]. Additional food categories were included to account for frequently consumed and culturally important foods for Māori and Pacific people, based on those identified by a Māori and Pacific expert panel [1]. The aggregated food categories were constructed in part to enable NOVA processing level [22] and fortification status to be coded, leading to a total of 61 food categories (Supplementary Table S1). Some foods were excluded as they were unable to be modeled as individual food categories (mixed dishes and takeaway foods), contributed negligible calories to the diet (tea and coffee, water, supplements), contributed negligible nutrients to the diet (alcohol), or were consumed infrequently (organ meats and offal, infant formula, baby foods). 

Three representative foods were selected within each food category, based on criteria used in the INFORMAS protocol [18]: most commonly consumed according to NZ nutrition survey data; culturally relevant to Māori and Pacific people; readily available in major NZ supermarkets; included in the NZFCDB (could be attributed to a unique food ID). For each food category, the mean (±SD) nutrient composition of the three representative foods, as sourced from the NZFCDB [21], was calculated.

#### 2.1.2. Food Price of Selected Representative Foods

The 2020 NRAUS Australia New Zealand Food Category Cost Dataset was created according to the INFORMAS protocol [18] and published elsewhere [23]. Food price data were obtained for each representative food, from two supermarkets (Countdown and Pak ‘n Save), located in both low (Hamilton Lake and Hamilton Central) and medium (Rototuna North and Rosa Birch Park) SES areas, within the Upper North Island of NZ, from 16 to 18 December 2020. The 2018 NZ Index of Deprivation (NZDep) [24] and SA2 areas were used for the selection of the low (Deciles 8–10) and medium (Deciles 4–6) SES locations.

The food price data were collected according to the following criteria: (i) the lowest non-discounted price was chosen; (ii) where more than one product size was available, the most commonly purchased size was chosen; (iii) the product was available nationally. Discounted fresh produce or that of poor quality (e.g., moldy, bruised, or damaged) was omitted, and if a specified representative product was not available, a similar product was selected, based on the nearest in nutrient composition (e.g., mandarins if oranges were not available). One food price sample was collected per each of the three representative food products, per each food category, SES location, and supermarket, leading to a total of 12 food price samples for each of the 61 food categories. The exception was for the ‘breakfast cereal, unfortified, sugars ≤15 g/100 g’ food category, for which only one representative product per supermarket was identified, leading to four food price samples for this food category. Cost information for each representative food was determined per 100 kcal, and the mean (±SD) cost per 100 kcal for each food category was calculated. All cost information is presented in NZ Dollars (NZD).

#### 2.1.3. Dietary Intake Database

To construct a current diet that provided an accurate representation of the NZ population, covering both low and medium SES areas, as well as Māori, Pacific, and NZEO population groups, dietary intake data were sourced from the 2008/2009 NZ Adult Nutrition Survey (ANS) [6], and the 2002 Children’s Nutrition Survey (CNS) [25], as Confidentialised Unit Record Files (CURFs) from Statistics NZ [26]. The 2008/2009 ANS was conducted using a computer-based interviewer-assisted three multiple-pass 24-h dietary recall method, with a total of 4721 participants completing the survey [6]. The 2002 CNS used a two-stage process that involved a random selection of 160 schools, followed by the random selection of children, with a total of 3275 participants [25]. The children gave their assent, and parents or guardians gave informed written consent for the study. The two (repeated) interviews for the collection of 24-h dietary recall data were primarily carried out at the child’s home in the presence of the parent/caregiver, using a direct computer data-entry system. Both surveys collected sociodemographic and anthropometric data. Where repeated 24-h recall data were recorded, data from the first day only were used in the current study to maximize sample size.

### 2.2. Classification of Each Food Category by NOVA Processing Level and Discretionary Status

Each of the 61 food categories was coded according to level of processing, as defined by the NOVA classification system [22,27,28], which organizes foods into four groups: Group 1, Unprocessed or minimally processed foods (e.g., meat, fish, milk, eggs, fruit, roots and tubers, vegetables, nuts and seeds, rice and other cereals); Group 2, Processed culinary ingredients (e.g., sugars, plant oils, and butter); Group 3, Processed foods (e.g., cheese, canned fruit and fish, smoked meats); Group 4, Ultra-processed foods (e.g., confectionaries, savory snacks, fast food dishes, ready-to-eat breakfast cereals, mass-produced packaged breads, and soft drinks). The NOVA processing level was coded for each food category based on previously published methodology applied to Australian foods [29], and conducted independently by two dietitians with expertise in the NOVA classification system and NZ foods. Each food category was further classified as discretionary or non-discretionary, based on the Australian Bureau of Statistics (ABS) discretionary food list (informed by the 2013 Australian Dietary Guidelines, which aligns with the NZ Eating and Activity Guidelines) [30]. Where a food category contained more than one processing level, or both discretionary and non-discretionary foods, the classification pertaining to the greatest number of foods within that food category was selected. 

### 2.3. Dietary Modeling Protocol

**Figure 1:**
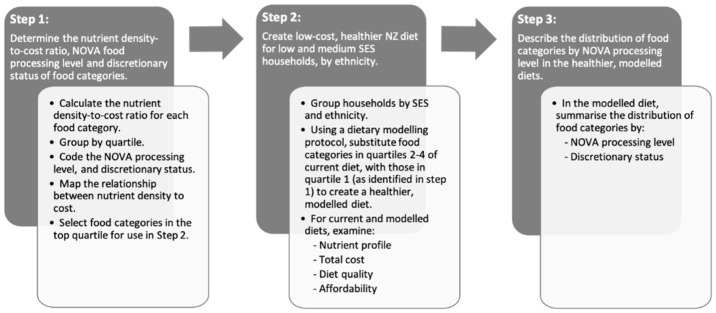
Summary of the major steps involved in the dietary modeling protocol to create nutrient dense, low-cost diets.

The dietary modeling protocol is summarized in Figure 1 and was based on a previously published Australian protocol [15], with adaptation to the NZ population and foods. 

Protocol involved three major steps: (1) determine the nutrient density-to-cost ratio of food categories; and their NOVA processing levels and discretionary status; (2) create low-cost, healthier NZ diets for low and medium SES households; and (3) determine the distribution of food categories by NOVA processing level in the healthier, modeled diets. 

The nutrient density-to-cost ratio for each food was calculated, via application of the nutrient rich food index (NRF9.3) [14,31], adapted for the NZ dietary guidelines and expressed per 100 kcal, followed by the division of NRF 9.3/100 kcal by cost in NZD/100 kcal (Supplementary Methods S1). A constraint that the ratio could not fall below zero was applied. The nutrient density-to-cost ratio for each food category was calculated as the mean of the ratios for the representative foods chosen in each food category; food categories in the highest quartile of nutrient density-to-cost ratio were considered to be ‘nutrient dense, low cost’ foods.

The top quartile of nutrient dense, low-cost food categories was used in substitution modeling to create low-cost, healthier NZ diets or Māori, Pacific, and NZEO populations from low and medium SES households. The household structure for all ethnic and SES populations was the four-person reference household recommended by the INFORMAS protocol [18], modified to cover the age ranges detailed in the 2008/2009 ANS and 2002 CNS, and to maximize the sample size for females 7–10 years, males 11–14 years, females 31–50 years, males 31–50 years. The characteristics of the six households modeled in the current study are provided in Table 1. Annual equivalized disposable income deciles for both low and medium socioeconomic households and for each population group were sourced from the 2019 NZ Household Income and Housing-Cost Statistics [32], and divided by 52 to determine the mean equivalized disposable income per week for each household. This was multiplied by the Organisation for Economic Co-operation and Development (OECD) adjustment factor to equivalize to the INFORMAS reference household [18].

Current diets for each household were modeled according to the 61 food categories, using survey dietary intake data [6,25] for each member of the reference household. Each food item in the dietary intake dataset was matched to one of the 61 food categories, based on its description, as well as its nutrient composition, as determined using the NZFCDB, and Food ID when available (for adult intake data only). The Food ID was not available for children’s data; thus, the description and nutrient composition were used only. Where a clear description of the food and/or nutrient composition data were unavailable, the match was made using the best judgement of the researchers. Daily intake data for each household member were summed to provide total household intakes. Weekly data were calculated by multiplying daily data by seven. For all substituted healthier more affordable diets, the aim was to align with the NZ dietary guideline recommendations for the servings per day of the core food groups (vegetables, fruit, grains, milk and milk products, and lean meat and alternatives) [33,34]. Core food group serving sizes were those stipulated in the NZ Eating and Activity guidelines for NZ adults, with additional information sourced from the Australian Eat for Health Educator’s Guide [34,35,36]. There was no allowance for discretionary foods, which were given a serving size equivalent to 150 kcal. Dairy milk alternatives were included in the milk and milk products core food group. 

The substitution rules used to develop the substitution modeling are described in Supplementary Methods S2. In brief, an algorithm was created that replaced food categories in the bottom three quartiles of nutrient density-to-cost ratio of the current diet with those in the highest quartile, according to the ‘like for like’ principle. Therefore, any substituted food was replaced with a more nutrient dense version of that food (e.g., full fat milk was replaced with reduced fat milk). The same principle was applied to discretionary foods. If a ‘like for like’ substitution using the top quartile was not available for core foods, the relevant top-ranking food category in the second quartile was used (e.g., there were no dairy or alternative foods in the top quartile; therefore, it was replaced with the top-ranking dairy or alternative from the second quartile). If it was not possible to make a ‘like for like’ substitution within the same core food group, the substitution was made using a more nutrient-dense, lower cost food category from the next most lacking core food group (e.g., dairy could be replaced with fruit). This was done according to a hierarchy, based on the highest proportion of individuals not meeting the dietary guidelines for that core food group within the current NZ diet, across both children and adults [6,25] as follows: fruit > vegetables > grains > dairy and alternatives > meat and alternatives. The algorithm was checked for logical output using key foods.

#### 2.3.1. Diet Quality

The diet quality of modeled diets was analyzed using validated diet quality indices (DQIs). As there were no validated NZ DQI for adults that scores diets based on compliance with the recommended serves of each core food group as detailed in the NZ Dietary Guidelines, the Healthy Eating Index for Australian Adults (HEIFA-2013) was selected as it aligned most closely to the NZ Dietary Guidelines [37]. The HEIFA-2013 was based on an 11-component system of five food groups (vegetables, fruits, grains, dairy and dairy alternatives, and meat and meat alternatives), three negative nutrients (fats, added sugars, and sodium), water intake and alcohol intake. Both current and healthier modeled diets were given the maximum (healthiest) score for component 7 (water) and component 11 (alcohol), as these foods were excluded from the current study.

The Dietary Index for Child’s Eating (DICE), a validated NZ DQI for children, was selected [38]. The DICE is based on a 13-component system (5 core food groups, fruit and vegetable variety, wholegrains, low fat/sugar/salt options, beverage selections, and eating patterns. Both current and healthier modeled diets were given the maximum (healthiest) score for water intake, tea and coffee intake, as these foods were excluded from the current study. A maximum score was also given for the eating patterns component, as this was not considered by the dietary modeling.

For both DQIs, a higher score indicated higher diet quality, with a maximum score of 100.

#### 2.3.2. Diet Cost and Affordability

The total cost of each modeled diet was calculated as the sum of the mean cost information (per serving size) for each food category included in the diet. Diet affordability was calculated by expressing the cost of each diet as a percentage of the OECD equivalized disposable income, adjusted to the INFORMAS reference household [18].

### 2.4. Statistical Analyses

Statistical analyses were performed using the R programming language (version 4.0.3, R Core Team, Vienna, Austria) [39], with the tidyverse packages (R studio, Boston, MA, USA) used extensively [40]. Population, nutrition, and cost input data were described by mean (SD), and dietary data produced from the substitution modeling protocol were presented as mean (SEM). Some of the analyses were carried out using SPSS (version 27, IBM, Armonk, NY, USA).

Statistical significance, for comparison of nutrition and cost between diets, was calculated via unpaired Students *t*-test, with adjustment using the False Discovery Rate Correction to account for multiple comparisons within each table. A general linear model for individuals’ change values (healthier—current) was constructed using ethnicity, SES and age-sex group, and all two-way interactions. This model was used to obtain statistical comparisons between diets for all SES and ethnic groups. *p*-values < 0.005 were considered statistically significant.

## 3. Results

### 3.1. Nutrient Density, Cost, and the Top Quartile of Nutrient Dense, Low-Cost NZ Food Categories

**Figure 2:**
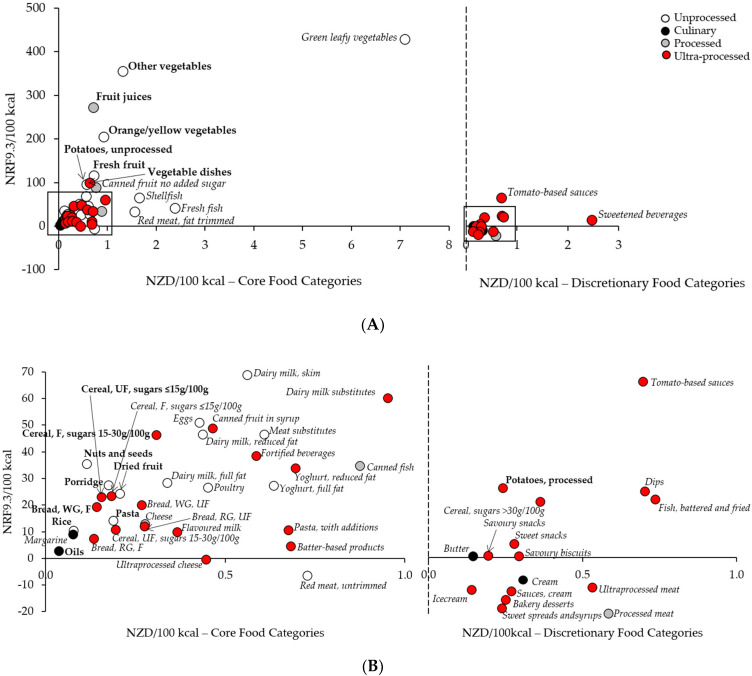
Nutrient density (NRF9.3 per 100 kcal) vs cost (NZD per 100 kcal) for the 61 NZ food categories (**A**) Complete graph showing the relationship between nutrient density and cost for all 61 food categories, separated as core or discretionary food categories. The majority of food categories are clustered with an NRF 9.3/100 kcal less than 70 and a cost (NZD)/100 kcal) less than NZD 1.0 (boxed areas). (**B**) Enlargement of the clustered food categories contained within the boxes in Figure 2A for each core and discretionary food category. For both A and B, the food categories appearing in the highest quartile of the nutrient density-to-cost ratio are shown in bold, and food categories in quartiles 2–4 are in italics. Unprocessed food categories are shown as white circles, culinary processed as black circles, processed as grey circles, and ultra-processed as red circles. Figure abbreviations: F, fortified; RTE, ready-to-eat; RG, refined grain; UF, unfortified; WG, wholegrain. Potatoes, unprocessed: includes unprocessed potatoes and red kumara; Potatoes, processed: includes processed potatoes and red kumara.

The nutrient density (NRF9.3/100 kcal) and cost (NZD/100 kcal) for the 61 food categories were plotted in Figure 2 and shown in Supplementary Table S2. Core and discretionary food categories were shown separately, on different sets of axes. Food category costs ranged from NZD 0.04/100 kcal (oils) to NZD 7.1/100 kcal (green leafy vegetables), and nutrient densities (NRF 9.3/100 kcal) from −20.3 (processed meat) to 428.5 (green leafy vegetables). All food categories with nutrient densities greater than 100/100 kcal were unprocessed fruit or vegetable-based core food categories, except for fruit juices which were processed and vegetable dishes (e.g., supermarket-deli coleslaw) which were ultra-processed (Figure 2A). The most nutrient dense discretionary food category was tomato-based sauces (NRF 9.3/100 kcal of 64.2). The majority of nutrient densities were clustered around a nutrient density score of less than 70/100 kcal for core food categories or 30/100 kcal for discretionary food categories, and a cost less than NZD 1/100 kcal for all food categories (Figure 2B). There was an even distribution of processing levels throughout all nutrient density and cost levels for core food categories, but there were no unprocessed discretionary food categories.

Foods that had the highest nutrient density for the lowest cost, based on the nutrient density-to-cost ratio (NRF 9.3/100 kcal)/(NZD/100 kcal), are shown in Table 2. The top quartile of nutrient density-to-cost food categories included foods from all core food groups except milk and milk products. The highest nutrient density-to-cost ratio was achieved by fruit juices (416.0), followed by other vegetables (337.9), and orange/yellow vegetables (324.3). Cereal and grain foods has the highest number of food categories score in the top quartile of nutrient density-to-cost ratio. There were no dairy or alternative foods in the top quartile of nutrient density-to-cost ratio; but there was one discretionary food (potatoes, red kumara, and taro, processed). The distribution of processing levels within the top quartile of nutrient dense, low-cost foods were 56% unprocessed, 31% ultra-processed, 6% processed, and 6% culinary processed. 

### 3.2. The Current NZ Diet for Māori, Pacific, and NZEO Households, from Low and Medium Socioeconomic Deciles

The current NZ diet was lacking in all core food groups across all households, for all ethnic populations, and both SES levels (Table 3). In contrast, discretionary food intake was high, with a range of 23.9 (NZEO, medium SES) to 36.9 (Māori, low SES) servings per week. The calculated household requirement for each of the core food groups and selected nutrients are shown in Supplementary Table S3.

The recommended acceptable macronutrient distribution range (AMDR) for protein of 15–25% energy was met by low SES Pacific households only [33]. Dietary fiber intake was low for all households (64.3 g/day to 75.8 g/day) compared to the summed minimum requirement of 100 g per household per day [33]. All households were within the AMDR for total fat of 20–35% energy, while saturated fat intake was above recommendations (range 11.0% to 12.3% of energy; recommended intake <10% energy). Added sugars were within recommendations (<10% energy) for both low (8.8%) and medium (10.0%) SES Pacific households, but higher for all other households (range 10.7% to 12.9%). Total carbohydrate intake was within the recommended range of 45–65% energy for all households. Intakes of micronutrients were consistently below summed requirements for the majority of households, except for thiamin, riboflavin, niacin, and vitamin B6 (Supplementary Table S4). In particular, calcium intake was approximately 50% of the household requirement value for all households. Sodium intake was high for some, but not all households, with low SES Māori (8858.8 mg/day), low SES Pacific (8522.3 mg/day), and medium SES Pacific (8532.0 mg/day) groups exceeding the summed maximum recommended household intake of 8450 mg/day (Supplementary Table S3) [33]. 

The cost of the current household diet ranged from NZD155.7 per week (NZEO low SES household) to NZD191.0 per week (Māori low SES household). On average, NZEO household diets were approximately 17.7% and 10.0% cheaper than the diets of Māori or Pacific households, for low and medium SES levels, respectively. Diet affordability in low SES households ranged from 19.3% to 23.6%, while medium SES households ranged from 10.4% to 11.9%.

### 3.3. The Theoretical Healthier, Low-Cost Diet

For all households, core food group intake increased in the healthier, low-cost diet, compared to the current diet (*p* < 0.005 for all), except for the lean meats, dairy, and their alternatives for Māori medium SES, and NZEO low and medium SES, in which there was no change (Table 4). Healthier diets met food group recommendations for fruit and grains only, except grains for NZEO low SES and Māori medium SES (Supplementary Table S3). The healthier, low-cost diet provided 63.7–77.7% of the recommended vegetable serves, 54.0–87.0% of the recommended lean meats and alternatives serves, and 34.3–51.4% of the recommended dairy and alternatives serves, depending on household.

Macronutrient intakes improved for all households, with decreased intakes of total fat, saturated fat, and added sugars (*p* < 0.001 for all) to achieve recommendations [33], and increased intakes of polyunsaturated fatty acids and dietary fiber (*p* < 0.001 for all). Dietary fiber intakes exceeded the recommended minimum 100g per day per household [33]. Protein intake remained below recommendations (range 13.1 to 13.8% of energy), [33], while total carbohydrates remained similar for all households except Pacific low SES. Total sugars also increased for some households (Table 4).

In low SES households, micronutrient intakes improved for vitamin A, B-group vitamins, vitamin E, calcium, iron, magnesium, potassium, and zinc (*p* ≤ 0.001 for all, except niacin for Pacific and NZEO), while vitamin B12 decreased (*p* < 0.001 for all). In medium SES households, micronutrient intakes improved for vitamin B6, folate, vitamin E, calcium, magnesium, potassium, and zinc (*p* < 0.001 for all, except calcium, potassium, folate, and zinc for Pacific; Supplementary Table S4). Sodium intakes decreased to below the recommended maximum intake for all households (*p* < 0.001 for all). While all healthier, low-cost diets met nutrient recommendations for most B vitamins, magnesium, and potassium, no household diet achieved the recommended dietary intake for vitamin A, B12, or calcium (Supplementary Tables S3 and S4).

The overall improvement in nutritional composition of the healthier diets (Table 4), compared to the current diets (Table 3), was accompanied by a decrease in total food costs for all households (*p* < 0.001 for all), of approximately 25.6% for low SES (range 22.7–27.3%), and 28.6% for medium SES (range 27.9–29.7%). This cost decrease persisted after expressing cost relative to energy intake (NZD/100 kcal) for all households (*p* < 0.001 for all; Table 4). Consistent with the pattern shown in the current diets, NZEO household diets were cheaper than the diets of Māori or Pacific households for low and medium SES levels (Table 4). Concurrent with the decrease in total food costs, total consumed food weight increased in the healthier diets for some households, compared to the current diet (Māori/Pacific low SES and NZEO medium SES; *p* < 0.005).

### 3.4. Diet Quality of the Current and Healthier Modeled Diets

**Figure 3:**
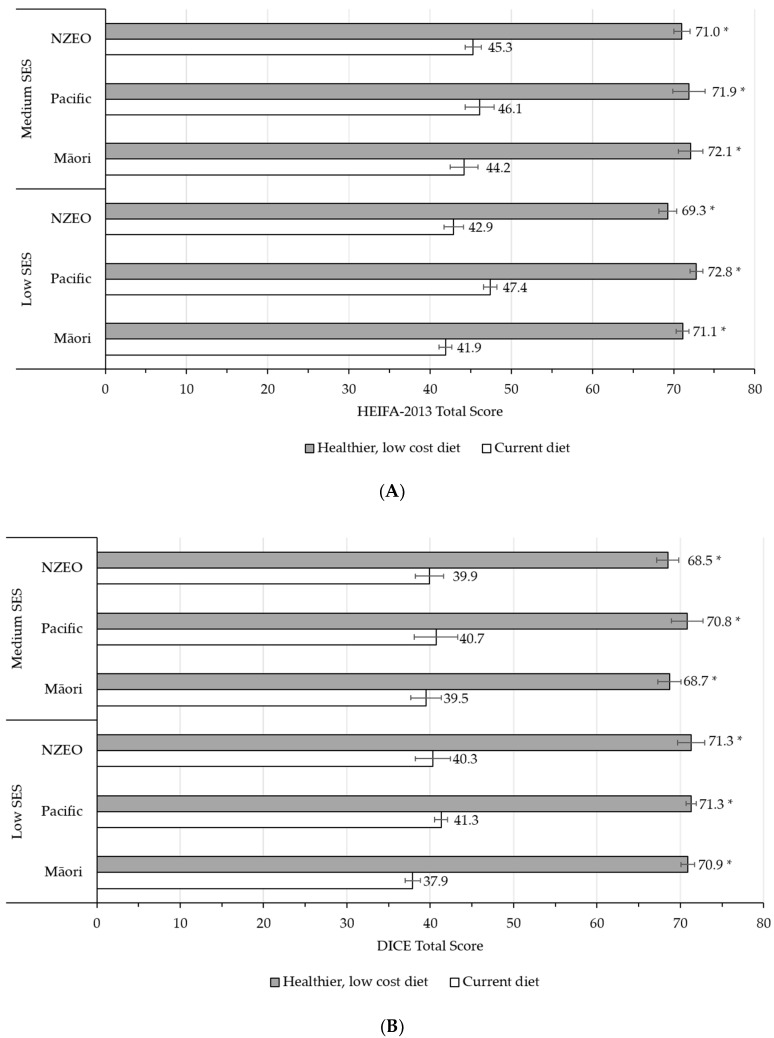
Diet quality of the current and healthier, low-cost diets, using validated diet quality indices. (**A**) HEIFA-2013 (Healthy Eating Index for Australian Adults) [37] assessment of the adult current and healthier, low-cost diets for Māori, Pacific, and NZEO low and medium SES households (**B**) DICE (Dietary Index for Child’s Eating) [38] assessment of the children’s current and healthier, low-cost diets for Māori, Pacific, and NZEO low and medium SES households. Statistical significance is denoted by * (*p* < 0.001); comparisons are between current and healthy diets within each group. SES, socioeconomic status.

Diet quality improved in the healthier, low-cost diet compared to the current diet for both adults and children of all ethnic groups and socioeconomic households. Adult diets improved from 41.9–47.4 to 69.3–72.8 (Figure 3A); and children’s diets improved from 37.9–41.3 to 68.5–71.3 (Figure 3B); *p* < 0.001 for all. Diet quality sub-scores are summarized in Supplementary Table S5. 

### 3.5. Distribution of NOVA Processing Levels and Food Categories in the Current and Healthier, Low-Cost Diets

All NOVA processing categories were represented in both current and healthier diets for all ethnic and SES groups (Table 5). In the current diets, ultra-processed foods contributed 59.2%, almost double that of unprocessed foods (32.0%), with 4.6% culinary processed foods and 4.3% processed foods. In the healthier, low-cost diets, the contribution of unprocessed foods more than doubled to 67.0% (*p* < 0.001 for all), with a decrease in ultra-processed foods to 25.9% (*p* < 0.001 for all). No differences in the percentage contribution of culinary processed foods or processed foods for most households were reported, except for a decrease in culinary processed foods for all Māori households (*p* < 0.001) and a decrease in processed foods for low SES Pacific and medium SES NZEO households (*p* < 0.005 for both; Table 5).

## 4. Discussion

This was the first study to determine the nutrient density-to-cost ratio of NZ foods and model the impact of substituting the current NZ diet with foods that were low cost and nutritious on diet quality and affordability by ethnicity and SES, with a secondary focus on food processing level. Findings suggest that the NZ diet can be made simultaneously healthier and more affordable, while containing a larger quantity of food for lower overall calories compared to the current diet for some households. When foods in the current NZ diet of Māori, Pacific, and NZEO households (low and medium SES) were replaced in a theoretical model with nutritious, lower cost alternatives, diet quality improved by 60.1% and 76.0% for adults and children, respectively, and affordability increased by 22.8–29.5%, depending on ethnicity and SES. While it is therefore possible to improve both diet quality and affordability simultaneously for these ethnic and SES groups in NZ, most core food group recommendations remained unmet in the healthier diets. This finding aligns with Australian data using a similar dietary modeling protocol [15]. In a previous NZ study [16], theoretical modeling created nutritionally complete, low-cost, and environmentally sustainable diets. However, the diets were largely based around unprocessed and minimally processed foods, thus omitting the nutrient-dense, low-cost ultra-processed foods identified in the current study, with no consideration for ethnic differences [16]. In remote NZ populations, access to fresh food can be limited due to geographical factors [42], suggesting that nutritious low-cost, ultra-processed foods such as vegetable dishes, wholegrain breads, and breakfast cereals with sugars at <30 g/100 g may play a valuable role in increasing diet quality for these communities. Research is needed to understand the lowest cost diet composition that achieves all food group and nutrient recommendations for all ethnic and age groups, while a greater understanding on how food security and sustainability efforts can be merged to best meet the unique needs of vulnerable populations in NZ.

No animal-derived food categories were in the top quartile of nutrient dense, low-cost food categories, including none from the dairy and alternatives core food group. Reflecting this, no families in the healthier, low-cost diets met nutrient targets for vitamin B12 (29.5% in adults and 22% in children inadequate) or calcium (39% in adults and 51% in children inadequate); and some failed to meet nutrient targets for zinc and potassium. Due to the provision of these key nutrients, NZ dietary guidelines recommend the inclusion of at least 2.5–3 servings of foods from the dairy and alternatives core food group per day [41]. While skim milk (bottled) emerged at the top of the second quartile of nutrient dense, low-cost foods, results confirm it is necessary to purchase foods that are relatively more expensive to meet food group recommendations. This finding is in contrast to Australian modeling data, which showed the top quartile of nutrient density-to-cost foods contained both reduced fat dairy milk and dairy milk alternatives, and represented all core food groups [15]. In Australia, there is no goods and services tax (GST) on healthy, basic foods such as fruit, vegetables, and milk; however, 10% GST is added to discretionary foods [1,17,43]. In NZ, 15% GST is added to all foods [44]. Research suggests if GST were to be removed from fruits, vegetables, and/or core foods in NZ, the affordability of a healthy NZ household diet would improve [1,17]. For example, if GST were removed from skim milk, the cost reduction (NZD 4.33 per 2L to NZD 3.76 per 2L) would move skim milk into the top quartile of the nutrient density-to-cost ratio and support access to healthy foods in NZ. These findings demonstrate a glass ceiling where the current NZ food environment may be unmanageable for families experiencing food stress, making it more difficult for them to choose affordable foods, adhere to dietary guidelines, and meet nutrient targets. Together, results may partially explain the current poor diet of the selected households and have implications for both nutrition guidelines and policies regarding food cost, taxation, and subsidies to improve the health of vulnerable NZ groups. 

Consistent with previous research that assessed the nutrient density-to-cost of foods in the USA [14,45] and Australia [15], all processing levels were represented in the top quartile of nutrient dense, low-cost food categories in NZ, supporting suggestions that some ultra-processed foods may be necessary for a healthy affordable diet [15,45,46,47]. Some of the ultra-processed food categories which were in the top quartile of nutrient density-to-cost ratio were also those in Australia and the USA, namely lower sugar (sugars <30 g/100 g) ready-to-eat breakfast cereals and wholegrain breads. Future research which examines the link between ultra-processed foods and NCDs should delineate these specific foods to reinform recommendations, so that blanket recommendations to avoid ultra-processed foods are not unnecessarily exclusionary to food-insecure population groups. 

The current Healthy Active Learning government initiative [48] provides Healthy Food and Drink Guidance for Schools [49], based on a traffic light system (green = consume frequently; amber = consume sometimes; red = avoid), but does not provide consideration for food costs. Ka Ora, Ka Ako (Live Well, Learn Well), which aims to deliver a free and healthy daily school lunch to Year 1-8 students in schools with high levels of disadvantage [50], seeks to align with the traffic light principles set out in the Ministry of Health Healthy Food and Drink Guidance for Schools [51], but could have been strengthened with attention to nutritious, low-cost foods. Additionally, Food Secure Communities, a 2-year program developed by the Ministry of Social Development in response to COVID-19 to support access to foodbanks and food rescue services [52], would benefit from information pertaining to the most nutrient-dense, low-cost foods.

While the top quartile of nutrient-dense, low-cost food categories found in this study largely align with the ‘green’ foods in the Healthy Food and Drink Guidance for Schools [49] and Ka Ora, Ka Ako nutrition guidelines [51], some were found to be expensive and therefore did not feature in the top quartile of nutrient density-to-cost ratio (e.g., leafy green vegetables and lean meats). In addition, some food categories included in the top quartile of nutrient density-to-cost ratio were ‘amber’ (e.g., dried fruit and fortified breakfast cereals with sugars between 15 and 30 g/100 g) and ‘red’ (e.g., fruit and vegetable juices containing 100% juice and no added sugars). Fruit juices are a core food whereby the NZ dietary guidelines recommend that fruit juices be limited to one serving (125 mL) per day [53,54]. Findings challenge their classification as ‘red’ (to be avoided), as they may provide an important source of nutrients for families in food stress. This is further strengthened by previous research which also reported fruit juices as the highest ranking food category according to the nutrient density-to-cost ratio [14,15] and a recent meta-analysis that reported a U-shaped curve showing protection against metabolic syndrome at moderate doses (125 mL per day) of 100% fruit juice [55]. While some RCT data suggest that 100% fruit juice could contribute to tooth erosion and dental caries in adults, findings are not supported in prospective cohort studies in children and adolescents [56]. While results in the current study are an artefact of the nutrient profiling tool chosen, which penalizes for added but not free sugars, evidence demonstrates the importance of choosing a tool to inform dietary recommendations that considers both nutritional quality and cost to minimize the barriers associated with equal access to healthy diets.

This study has several strengths. It provided important information about which foods have the highest nutrient profile for the lowest cost in NZ, using the NRF 9.3, a validated tool for the assessment of nutritional quality and nutrient density [31]. This list can be compared to similar lists ranking the nutrient density-to-cost of food categories, produced for Australia and the USA, to allow insight into global differences in food prices. The data used in the substitution modeling was nationally representative, and provided novel examination of differences across major ethnic populations and SES groups in NZ. The limitations of this study are primarily related to the input data used to inform the substitution modeling methodology. The most recent NZ Adult and Children’s Nutrition Surveys were carried out in 2008/2009 and 2002, respectively. The generalizability of findings may be limited by the age of the data, the impact of recent dietary trends and reformulation efforts by food manufacturers [57]. The NRF9.3 does not consider all nutrients and is particularly limited by the exclusion of B group vitamins which has implications for the top quartile of nutrient density-to-cost findings. However, all families met the recommended targets for B-group vitamins, except for vitamin B12. This study was intended as a theoretical proof of concept only. It was not possible to model the entire NZ diet, as only foods available in two major NZ supermarket chains were included, with foods such as takeaways and mixed meals excluded. Findings may not be applicable to low SES households living in remote locations that do not have ready access to supermarkets and that purchase the majority of food from local dairies (small, independent providers of key products, containing a smaller range sold at a higher cost, compared to supermarkets) and takeaway outlets. While food categories were designed to include ethnically relevant foods such as taro, coconut cream, and watercress, these foods were not modeled individually. Findings may not be translatable to individual dietary advice, particularly for Māori and Pacific populations consuming an ethnic diet. Further research is required to understand the impact of nutrient dense, low-cost foods on the diet of specific ethnic populations and those with poor access to supermarkets. Lastly, although results suggest some differences between ethnicities with respect to diet cost and composition, these differences were not assessed statistically and require further investigation.

## 5. Conclusions

Nutrient dense, low-cost core foods can make NZ diets healthier and more affordable, while having a positive impact on energy density for Māori, Pacific, and NZEO households from both low and medium SES areas. While most nutrient dense, low-cost core foods were predominantly unprocessed, there was a notable contribution of ultra-processed food which suggests some non-discretionary, ultra-processed foods may provide a beneficial source of low-cost nutrition for food insecure populations. Future research is needed to examine findings in remote populations and to determine if a diet containing nutrient-dense, low-cost foods can be modeled to meet national dietary recommendations, whilst embracing the complexity of all foods consumed by families.

## Figures and Tables

**Table 1:** Characteristics of low and medium SES reference households, for Māori, Pacific, and NZEO ethnic groups, as well as the total population.

Indicators ^1^	Low SES Household				Medium SES Household			
	Māori	Pacific	NZEO	Total	Māori	Pacific	NZEO	Total
	4-person reference household				4-person reference household			
**Household member ^2^**								
**Male (31–50 years)**								
*n*	70	81	39	190	20	14	54	88
Age (y)	39.5 (5.4)	39.8 (4.8)	38.9 (5.7)	39.5 (5.2)	42.0 (6.0)	39.4 (6.9)	40.0 (6.0)	40.4 (6.1)
Height (cm)	174.1 (6.5)	175.2 (5.5)	175.8 (8.7)	174.9 (6.6)	176.5 (7.4)	177.6 (7.0)	176.8 (6.6)	176.8 (6.8)
Weight (kg)	95.9 (22.6)	105.1 (20.1)	82.3 (16.3)	97.0 (22.0)	96.4 (16.5)	109.1 (22.3)	85.4 (14.0)	91.7 (18.3)
BMI (kg/m^2^)								
Underweight (<18.5), *n* (%)	0 (0.0%)	1 (1.3%)	1 (2.6%)	2 (1.1%)	0 (0.0%)	0 (0.0%)	0 (0.0%)	0 (0.0%)
Normal (18.5–24.9), *n* (%)	8 (12.1%)	3 (3.7%)	15 (38.5%)	26 (14.0%)	1 (5.3%)	1 (7.1%)	14 (26.9%)	16 (18.8%)
Overweight (25.0–29.9), *n* (%)	28 (42.4%)	15 (18.8%)	16 (41.0%)	59 (31.9%)	7 (36.8%)	2 (14.3%)	27 (51.9%)	36 (42.4%)
Obese (≥30.0), *n* (%)	30 (45.5%)	61 (76.2%)	7 (17.9%)	98 (53.0%)	11 (57.9%)	11 (78.6%)	11 (21.2%)	33 (38.8%)
**Female (31–50 years)**								
*n*	128	96	52	276	29	19	67	115
Age (y)	38.9 (5.5)	39.2 (5.4)	40.4 (5.9)	39.3 (5.6)	38.9 (5.7)	40.6 (4.9)	40.7 (5.5)	40.2 (5.5)
Height (cm)	163.5 (5.2)	163.9 (5.3)	161.5 (6.0)	163.3 (5.5)	162.8 (5.6)	164.6 (3.9)	163.8 (6.9)	163.7 (6.1)
Weight (kg)	89.0 (24.4)	95.4 (22.0)	74.4 (17.6)	88.3 (23.5)	80.1 (18.7)	94.2 (18.7)	76.2 (17.0)	80.2 (18.8)
BMI (kg/m^2^)								
Underweight (<18.5), *n* (%)	1 (0.9%)	0 (0.0%)	0 (0.0%)	1 (0.4%)	0 (0.0%)	0 (0.0%)	0 (0.0%)	0 (0.0%)
Normal (18.5–24.9), *n* (%)	18 (15.5%)	4 (4.6%)	13 (26.0%)	35 (13.8%)	5 (17.2%)	1 (5.3%)	21 (32.3%)	27 (23.9%)
Overweight (25.0–29.9), *n* (%)	27 (23.3%)	22 (25.3%)	23 (46.0%)	72 (28.5%)	15 (51.7%)	4 (21.0%)	23 (35.4%)	42 (37.2%)
Obese (≥30.0), *n* (%)	70 (60.3%)	61 (70.1%)	14 (28.0%)	145 (57.3%)	9 (31.0%)	14 (73.7%)	21 (32.3%)	44 (38.9%)
**Male (11–14 years)**								
*n*	67	110	17	194	36	13	46	95
Age (y)	12.4 (1.2)	12.4 (1.1)	12.5 (0.9)	12.4 (1.1)	12.4 (1.2)	12.5 (1.1)	12.4 (1.1)	12.4 (1.2)
Height (cm)	162.7 (11.6)	161.7 (11.2)	159.6 (8.5)	161.9 (11.1)	160.6 (11.6)	160.5 (10.3)	158.6 (11.6)	159.6 (11.3)
Weight (kg)	58.4 (19.4)	65.4 (21.0)	56.3 (11.5)	62.2 (20.1)	55.0 (15.6)	58.1 (14.8)	53.8 (12.8)	54.8 (14.1)
BMI (kg/m^2^)								
Underweight (<18.5), *n* (%)	0 (0.0%)	0 (0.0%)	0 (0.0%)	0 (0.0%)	0 (0.0%)	0 (0.0%)	0 (0.0%)	0 (0.0%)
Normal (18.5–24.9), *n* (%)	43 (68.3%)	40 (38.1%)	8 (53.3%)	91 (49.7%)	21 (65.6%)	4 (33.3%)	27 (62.8%)	52 (59.8%)
Overweight (25.0–29.9), *n* (%)	12 (19.0%)	33 (31.4%)	6 (40.0%)	51 (27.9%)	8 (25.0%)	6 (50.0%)	13 (30.2%)	27 (31.0%)
Obese (≥30.0), *n* (%)	8 (12.7%)	32 (30.5%)	1 (6.7%)	41 (22.4%)	3 (9.4%)	2 (16.7%)	3 (7.0%)	8 (9.2%)
**Female (7–10 years)**								
*n*	127	133	25	285	31	20	24	75
Age (y)	8.4 (1.2)	8.6 (1.1)	8.6 (1.2)	8.5 (1.1)	8.3 (1.2)	8.6 (1.2)	8.6 (1.2)	8.5 (1.2)
Height (cm)	135.6 (9.8)	139.4 (9.9)	135.1 (7.2)	137.4 (9.8)	136.4 (11.9)	137.5 (10.2)	135.0 (9.4)	136.3 (10.6)
Weight (kg)	36.5 (11.0)	41.9 (13.0)	32.9 (6.5)	38.9 (12.1)	37.3 (13.8)	37.3 (10.9)	35.1 (9.4)	36.6 (11.7)
BMI (kg/m^2^)								
Underweight (<18.5), *n* (%)	0 (0.0%)	0 (0.0%)	0 (0.0%)	0 (0.0%)	0 (0.0%)	0 (0.0%)	0 (0.0%)	0 (0.0%)
Normal (18.5–24.9), *n* (%)	59 (50.9%)	50 (38.7%)	17 (73.9%)	126 (47.0%)	14 (46.7%)	9 (45.0%)	12 (54.5%)	35 (48.6%)
Overweight (25.0–29.9), *n* (%)	35 (30.2%)	34 (26.4%)	4 (17.4%)	73 (27.2%)	9 (30.0%)	9 (45.0%)	8 (36.4%)	26 (36.1%)
Obese (≥30.0), *n* (%)	22 (19.0%)	45 (34.9%)	2 (8.7%)	69 (25.7%)	7 (23.3%)	2 (10.0%)	2 (9.1%)	11 (15.3%)
**SES Data**								
Decile of socioeconomic deprivation (NZDep) ^3^	9–10				5–6			
Equivalised disposable household income ^4^	$404/week				$775/week			
Adjusted household income ^5^	$808/week				$1550/week			

^1^ All data are mean (SD) of all data used for analysis, unless otherwise stated. BMI data and categories were taken directly from the relevant survey microdata. ^2^ Four-person reference household, as defined by the INFORMAS protocol [18], adapted to align with the age groups used in the 2008/2009 Adult Nutrition Survey (ANS) [6], and the 2002 Children’s Nutrition Survey (CNS) [25]. ^3^ Defined according to the 2018 NZ Index of Deprivation (NZDep) [24]. ^4^ Sourced from the 2019 NZ Household Income and Housing-Cost Statistics [32]. ^5^ Equivalized disposable income (2017–2018) multiplied by the OECD adjustment factor of 2 (for a household of 4) [18]. BMI, body mass index; SES, socioeconomic status; NZEO, New Zealand European and Other.

**Table 2:** The top quartile of nutrient dense, low-cost food categories. NRF 9.3/100 kcal, NZD/100 kcal, nutrient density-to-cost ratio, and NOVA processing level of each food category are shown.

Food Sub-Groups ^1^	NRF 9.3/100 kcal	$/100 kcal	Nutrient Density-to-Cost Ratio ^2^	Processing Level
**Vegetables**				
Other vegetables	355.7 (162.8)	1.3 (0.8)	337.9 (179.9)	Unprocessed
Orange/yellow vegetables	205.3 (88.1)	0.9 (0.3)	324.3 (335.0)	Unprocessed
Potatoes and red kumara, unprocessed	96.4 (19.9)	0.6 (0.3)	196.1 (77.7)	Unprocessed
Vegetable dishes	99.7 (90.8)	0.6 (0.4)	183.2 (192.9)	Ultra-processed
**Fruit**				
Fruit juices	272.2 (105.8)	0.7 (0.3)	416.0 (102.7)	Processed
Fresh fruit	116.6 (106.8)	0.7 (0.4)	159.1 (84.2)	Unprocessed
Dried fruit	24.3 (4.2)	0.2 (0.1)	126.8 (46.0)	Unprocessed
**Cereals and grain foods**				
Hot porridge	27.5 (9.8)	0.2 (0.1)	246.0 (153.6)	Unprocessed
Pasta and noodles	14.2 (6.8)	0.2 (0.2)	180.9 (137.1)	Unprocessed
Breads and rolls, wholegrain, fortified	19.4 (2.2)	0.1 (0.0)	150.5 (55.1)	Ultra-processed
Breakfast cereal, unfortified, sugars ≤15 g/100 g	23.1 (0.0)	0.2 (0.0)	148.9 (3.5)	Ultra-processed
Breakfast cereal, fortified, sugars 15–30 g/100 g	46.4 (19.8)	0.3 (0.0)	148.4 (58.4)	Ultra-processed
Rice and grains	10.5 (6.3)	0.1 (0.0)	136.4 (70.3)	Unprocessed
**Meat and alternatives**				
Nuts and seeds	35.5 (11.8)	0.1 (0.0)	303.6 (50.7)	Unprocessed
**Other foods**				
Oils	2.9 (4.8)	0.04 (0.0)	138.5 (103.9)	Culinary processed
**Discretionary foods**				
Potatoes and red kumara, processed	25.4 (10.3)	0.2 (0.1)	129.2 (81.4)	Ultra-processed

^1^ Data are mean (SD). ^2^ The nutrient density-to-cost ratio for a food category is the mean of the individual ratios of the representative foods within each food category. NRF, nutrient rich foods index.

**Table 3:** The current NZ diet for each reference household, by ethnicity and SES.

**Current Diet ^1^**	**Low SES Household**			**Medium SES Household**		
	**Māori**	**Pacific**	**NZEO**	**Māori**	**Pacific**	**NZEO**
*n*	391	420	133	116	66	190
Total energy (kcal/day)	7286.1 (111.5)	7275.2 (103.4)	6228.2 (126.4)	6260.3 (159.6)	6931.1 (236.3)	6247.9 (130.5)
**Food groups (servings/day) ^2^**						
Vegetables (21.5 servings/day)	7.0 (0.3)	10.0 (0.4)	6.6 (0.4)	6.3 (0.5)	9.2 (0.8)	7.8 (0.4)
Fruit (8 servings/day)	4.4 (0.2)	5.5 (0.2)	5.1 (0.3)	6.6 (0.3)	5.4 (0.4)	5.9 (0.2)
Grains and cereals (22 servings/day)	11.9 (0.4)	15.5 (0.4)	11.8 (0.5)	10.9 (0.5)	13.3 (0.9)	14.4 (0.6)
Lean meats and alternatives (10 servings/day)	5.5 (0.2)	6.8 (0.2)	3.9 (0.3)	5.2 (0.4)	5.5 (0.4)	3.6 (0.2)
Dairy and alternatives (10.5 servings/day)	3.1 (0.1)	2.2 (0.1)	3.5 (0.2)	3.4 (0.2)	2.7 (0.2)	3.5 (0.1)
Discretionary foods (<11 servings/day)	36.9 (0.8)	31.6 (0.8)	27.3 (0.9)	27.4 (1.3)	28.2 (1.6)	23.9 (0.7)
**Macronutrients (% of energy) ^3^**						
Protein (15–25%)	14.2 (0.2)	14.9 (0.2)	13.1 (0.3)	13.2 (0.3)	14.1 (0.4)	13.1 (0.2)
Total fat (<35%)	34.3 (0.3)	31.5 (0.3)	30.3 (0.4)	31.6 (0.4)	31.7 (0.7)	28.7 (0.4)
Saturated fat (<10%)	12.3 (0.1)	11.0 (0.1)	11.7 (0.2)	11.7 (0.2)	11.7 (0.3)	11.2 (0.2)
Monounsaturated fat	12.6 (0.1)	11.6 (0.1)	10.1 (0.2)	11.0 (0.2)	11.5 (0.3)	9.6 (0.2)
Polyunsaturated fat	5.5 (0.1)	5.4 (0.1)	5.1 (0.1)	5.2 (0.1)	5.0 (0.1)	4.7 (0.1)
Total Carbohydrates (45–65%)	49.7 (0.3)	51.5 (0.3)	54.6 (0.5)	53.2 (0.5)	52.3 (0.8)	56.1 (0.4)
Total sugars	20.0 (0.3)	19.0 (0.3)	24.4 (0.5)	24.5 (0.6)	20.1 (0.7)	22.7 (0.4)
Added sugars (<10%)	10.7 (0.2)	8.8 (0.2)	12.9 (0.4)	12.0 (0.5)	10.0 (0.5)	11.2 (0.3)
Free sugars	12.2 (0.2)	10.2 (0.2)	14.6 (0.5)	14.4 (0.5)	11.2 (0.5)	13.0 (0.3)
Starch	29.5 (0.3)	32.3 (0.3)	29.9 (0.5)	28.5 (0.5)	32.0 (0.8)	33.2 (0.5)
Dietary fiber (g/day) ^4^ (100 g/day)	67.6 (1.2)	75.8 (1.2)	65.9 (1.8)	64.3 (1.7)	70.8 (2.7)	69.6 (1.5)
**Cost and affordability**						
Cost of diet (NZD/week)	191.0 (3.3)	187.3 (2.9)	155.7 (3.3)	173.8 (6.0)	184.9 (6.5)	161.3 (3.5)
Diet affordability (%) ^5^	23.6	23.2	19.3	11.2	11.9	10.4
Diet energy cost (NZD/100 kcal)	2.7 (0.0)	2.7 (0.0)	2.6 (0.0)	2.8 (0.1)	2.7 (0.1)	2.6 (0.0)
**Total consumed food (g/day)**	5131.5 (72.1)	5242.6 (71.7)	4644.8 (94.8)	4538.0 (98.3)	5040.0 (168.4)	4534.8 (77.8)

^1^ All data are mean (SEM) per day of the combination of average data for each household (i.e., adult male + adult female + child male + child female) for all diets used in the analyses. ^2^ Servings per day were calculated based on the recommendations provided in the updated Ministry of Health serving size advice sheet [41]. The recommended number of household servings, taken as the sum of the recommended number of servings for each household member (Supplementary Table S3) is shown in brackets for each core food group. ^3^ The recommended dietary intake, as a percentage of energy, shown in brackets for relevant macronutrients. ^4^ The recommended daily intake of dietary fiber for a household was taken as the sum of the recommended intakes for each household member (Supplementary Table S3) and is shown in brackets as 100g/day. ^5^ Diet affordability is the mean diet cost per week expressed as a percentage of equivalized disposable income (as shown in Table 1). NZEO, New Zealand European and other ethnic groups; SES, Socioeconomic Status.

**Table 4:** The theoretical modeled healthier, low-cost NZ diet for each reference household, by ethnicity and SES.

Healthier Modeled Diet ^1,2^	Low SES Household			Medium SES Household			Statistical Comparisons (*p*-Value) ^3^					
							Current vs. Healthier Diet Low SES			Current vs. Healthier Diet Medium SES		
	Maori	Pacific	NZEO	Maori	Pacific	NZEO	Maori	Pacific	NZEO	Maori	Pacific	NZEO
*n*	391	420	133	116	66	190						
Total energy (kcal/day)	7717.6 (131.2)	7494.8 (105.7)	6087.1 (131.8)	6148.4 (165.8)	6830.3 (232.7)	6189.2 (125.8)	<0.011	<0.001	<0.001	0.030	<0.001	<0.001
**Food groups (servings/day)**												
Vegetables (21.5 servings/day)	14.9 (0.4)	16.2 (0.4)	13.7 (0.6)	14.9 (0.8)	16.7 (1.2)	13.7 (0.5)	<0.001	<0.001	<0.001	<0.001	<0.001	<0.001
Fruit (8 servings/day)	12.6 (0.3)	13.9 (0.4)	11.9 (0.4)	13.4 (0.5)	11.9 (0.5)	11.4 (0.4)	<0.001	<0.001	<0.001	<0.001	<0.001	<0.001
Grains and cereals (22 servings/day)	26.7 (0.6)	28.1 (0.6)	21.5 (0.6)	19.9 (0.7)	23.4 (1.0)	23.6 (0.7)	<0.001	<0.001	<0.001	<0.001	<0.001	<0.001
Lean meats and alternatives (10 servings/day)	8.0 (0.3)	8.7 (0.2)	5.4 (0.3)	6.8 (0.4)	7.6 (0.5)	5.4 (0.2)	0.001	0.017	0.061	0.869	0.017	0.694
Dairy and alternatives (10.5 servings/day)	5.4 (0.3)	3.6 (0.2)	5.1 (0.3)	4.0 (0.2)	4.1 (0.4)	4.4 (0.2)	<0.001	<0.001	0.001	0.113	<0.001	0.010
Discretionary foods (<11 servings/day)	0.0 (0.0)	0.0 (0.0)	0.0 (0.0)	0.0 (0.0)	0.0 (0.0)	0.0 (0.0)	<0.001	<0.001	<0.001	<0.001	<0.001	<0.001
**Macronutrients (% of energy)**												
Protein (15–25%)	13.7 (0.1)	13.2 (0.1)	13.8 (0.2)	13.1 (0.1)	13.2 (0.2)	13.7 (0.1)	<0.001	<0.001	0.845	0.179	0.001	0.767
Total fat (<35%)	25.7 (0.4)	25.8 (0.3)	21.6 (0.5)	22.9 (0.6)	23.5 (0.8)	20.7 (0.5)	<0.001	<0.001	<0.001	<0.001	<0.001	<0.001
Saturated fat (<10%)	4.2 (0.1)	4.2 (0.0)	3.7 (0.1)	3.8 (0.1)	3.9 (0.1)	3.6 (0.1)	<0.001	<0.001	<0.001	<0.001	<0.001	<0.001
Monounsaturated fat	11.2 (0.2)	11.4 (0.2)	9.1 (0.3)	9.9 (0.3)	10.4 (0.4)	8.8 (0.2)	<0.001	<0.001	<0.001	0.048	<0.001	0.001
Polyunsaturated fat	8.7 (0.1)	8.7 (0.1)	7.3 (0.2)	7.7 (0.2)	7.8 (0.3)	7.0 (0.2)	<0.001	<0.001	0.012	<0.001	0.007	0.001
Total Carbohydrates (45–65%)	56.4 (0.4)	56.8 (0.3)	60.4 (0.5)	59.8 (0.6)	59.2 (0.8)	61.5 (0.5)	0.358	<0.001	0.714	0.036	0.578	0.913
Total sugars	23.1 (0.3)	22.4 (0.3)	25.9 (0.5)	27.1 (0.7)	22.3 (0.7)	23.4 (0.4)	<0.001	<0.001	0.019	0.003	0.001	0.010
Added sugars (<10%)	1.9 (0.1)	1.9 (0.1)	2.0 (0.1)	1.7 (0.1)	1.6 (0.1)	1.9 (0.1)	<0.001	<0.001	<0.001	<0.001	<0.001	<0.001
Free sugars	2.9 (0.1)	2.7 (0.1)	2.9 (0.1)	3.7 (0.4)	2.3 (0.2)	2.9 (0.1)	<0.001	<0.001	<0.001	<0.001	<0.001	<0.001
Starch	32.9 (0.4)	34.1 (0.3)	34.2 (0.5)	32.4 (0.6)	36.6 (0.9)	37.8 (0.6)	<0.001	<0.001	0.008	0.424	<0.001	0.014
Dietary fiber (g/day) (100 g/day)	173.0 (3.1)	169.9 (2.6)	136.8 (3.2)	141.8 (4.1)	152.4 (5.9)	137.4 (3.1)	<0.001	<0.001	<0.001	<0.001	<0.001	<0.001
**Cost and affordability**												
Cost of diet (NZD/week)	138.9 (2.4)	137.3 (2.2)	120.3 (2.7)	122.1 (3.5)	133.0 (5.2)	116.3 (2.3)	<0.001	<0.001	<0.001	<0.001	<0.001	<0.001
Diet affordability (%) ^4^	17.2	17.0	14.9	7.9	8.6	7.5						
Diet energy cost (NZD/100 kcal)	2.0 (0.0)	1.9 (0.0)	2.1 (0.0)	2.1 (0.0)	2.0 (0.0)	2.0 (0.0)	<0.001	<0.001	<0.001	<0.001	<0.001	<0.001
Total consumed food (g/day)	5736.4.1 (120.1)	5661.1 (96.4)	5153.7 (122.4)	4891.1 (140.7)	5535.5 (220.5)	4961.3 (98.6)	<0.001	0.001	0.007	0.076	0.202	0.003

^1^ All data are mean (SEM) per day of the combination of average data for each household (i.e., adult male + adult female + child male + child female) for all diets used in the analyses. ^2^ Recommended dietary intakes, taken as the sum of the recommended daily intake for each household member, are shown in brackets and were calculated based on the recommendations provided in the updated Ministry of Health serving size advice sheet [41]. ^3^ Comparison between the current vs healthier diet for each low SES and medium SES ethnic population group. For statistical significance, *p* < 0.005. ^4^ Diet affordability is the mean diet cost per week expressed as a percentage of equivalized disposable income (as shown in Table 1). NZEO, New Zealand European and other ethnic groups; SES, Socioeconomic Status.

**Table 5:** Distribution of NOVA processing levels and food category types throughout the current and healthier diets for each ethnic group, and both low and medium SES households.

Category Distribution ^1^	Low SES Household						Medium SES Household						Statistical Comparisons (*p*-Value) ^2^					
	Māori		Pacific		NZEO		Māori		Pacific		NZEO		Current vs. Healthier Diet Low SES			Current vs. Healthier Diet Medium SES		
	C	H	C	H	C	H	C	H	C	H	C	H	M	P	N	M	P	N
**Unprocessed**	27.7% (1.1)	65.5% (1.3)	35.7% (1.2)	65.1% (1.2)	31.4% (2.3)	66.7% (2.3)	29.0% (2.1)	65.7% (2.1)	34.9% (2.7)	71.4% (2.9)	33.2% (1.8)	67.8% (1.6)	<0.001	<0.001	<0.001	<0.001	<0.001	<0.001
Potatoes and red kumara	13.2% (1.3)	13.0% (1.0)	13.7% (1.2)	11.6% (0.9)	9.4% (1.9)	7.5% (1.3)	11.8% (2.1)	13.1% (1.7)	19.7% (3.4)	17.0% (2.5)	12.1% (1.9)	10.7% (1.3)	0.816	0.047	0.317	0.360	0.332	0.576
Orange/yellow vegetables	1.8% (0.4)	4.6% (0.5)	0.9% (0.2)	4.2% (0.5)	3.0% (0.8)	5.6% (1.1)	1.2% (0.4)	6.0% (1.4)	1.4% (0.8)	4.6% (1.6)	2.5% (0.6)	3.8% (0.6)	<0.001	<0.001	0.015	<0.001	0.028	0.232
Green leafy vegetables	1.3% (0.4)	0.0% (0.0)	0.8% (0.3)	0.0% (0.0)	0.6% (0.3)	0.0% (0.0)	0.8% (0.4)	0.0% (0.0)	0.7% (0.4)	0.0% (0.0)	0.9% (0.3)	0.0% (0.0)	<0.001	0.010	0.313	0.215	0.424	0.066
Other vegetables	6.1% (0.9)	6.8% (0.7)	11.7% (1.2)	9.8% (0.8)	8.4% (1.7)	7.5% (1.3)	4.8% (1.0)	6.2% (1.0)	5.6% (1.6)	5.8% (1.1)	7.8% (1.1)	8.5% (1.1)	0.354	0.040	0.849	0.332	0.893	0.480
Fresh fruit	19.7% (1.5)	17.3% (1.0)	19.8% (1.3)	20.2% (1.1)	23.9% (3.2)	22.7% (2.5)	26.8% (3.0)	20.8% (2.1)	27.0% (3.8)	21.4% (2.8)	25.0% (2.6)	17.6% (1.5)	0.144	0.816	0.616	0.034	0.095	<0.001
Dried fruit	0.4% (0.4)	10.0% (0.9)	0.0% (0.0)	8.7% (0.9)	1.5% (0.9)	9.9% (1.5)	1.7% (0.8)	12.6% (1.9)	0.2% (0.2)	5.0% (1.4)	0.6% (0.3)	9.0% (1.3)	<0.001	<0.001	<0.001	<0.001	0.032	<0.001
Rice and grains	3.3% (0.7)	5.1% (0.7)	11.3% (1.1)	9.1% (0.8)	12.2% (2.2)	10.7% (1.4)	1.4% (0.7)	6.1% (1.4)	11.4% (3.6)	9.4% (2.3)	11.6% (2.1)	13.8% (1.8)	0.052	0.009	0.626	0.009	0.484	0.075
Pasta, plain	1.0% (0.5)	4.8% (0.7)	0.7% (0.3)	4.2% (0.7)	2.5% (1.0)	3.0% (0.7)	1.2% (0.7)	2.5% (0.8)	0.3% (0.3)	7.5% (2.0)	3.0% (1.5)	4.8% (1.0)	<0.001	<0.001	0.997	0.410	<0.001	0.180
Hot porridge	1.3% (0.4)	7.4% (0.9)	0.8% (0.3)	4.4% (0.6)	1.7% (1.4)	6.0% (1.3)	0.5% (0.4)	6.5% (1.4)	0.6% (0.6)	4.8% (1.8)	1.0% (0.5)	6.0% (1.0)	<0.001	<0.001	0.001	<0.001	0.132	<0.001
Dairy milk full/reduced fat	22.4% (1.7)	0.0% (0.0)	12.4% (1.1)	0.0% (0.0)	15.2% (2.4)	0.0% (0.0)	22.3% (3.1)	0.0% (0.0)	14.0% (2.8)	0.0% (0.0)	16.1% (2.1)	0.0% (0.0)	<0.001	<0.001	<0.001	<0.001	<0.001	<0.001
Dairy milk skim	0.7% (0.3)	11.4% (0.8)	0.9% (0.4)	8.6% (0.5)	2.1% (0.8)	12.8% (1.2)	1.1% (0.5)	10.7% (1.2)	0.2% (0.1)	9.6% (2.1)	2.6% (0.9)	11.8% (1.0)	<0.001	<0.001	<0.001	<0.001	<0.001	<0.001
Yoghurt full fat	1.1% (0.3)	0.0% (0.0)	1.2% (0.3)	0.0% (0.0)	0.5% (0.2)	0.0% (0.0)	2.5% (0.9)	0.0% (0.0)	0.2% (0.1)	0.0% (0.0)	1.3% (0.5)	0.0% (0.0)	0.008	0.002	0.500	<0.001	0.888	0.037
Nuts and seeds	1.6% (0.4)	19.5% (1.2)	2.2% (0.4)	19.0% (0.9)	3.0% (1.1)	14.2% (1.7)	3.0% (1.2)	15.5% (1.6)	0.6% (0.3)	14.8% (1.7)	2.6% (0.6)	14.0% (1.2)	<0.001	<0.001	<0.001	<0.001	<0.001	<0.001
Fish, meat, eggs and poultry	26.1% (1.7)	0.0% (0.0)	23.7% (1.3)	0.0% (0.0)	15.9% (2.2)	0.0% (0.0)	20.8% (2.7)	0.0% (0.0)	18.1% (3.0)	0.0% (0.0)	12.8% (1.7)	0.0% (0.0)	<0.001	<0.001	<0.001	<0.001	<0.001	<0.001
**Processed culinary**	5.7% (0.5)	5.4% (0.4)	5.5% (0.4)	5.3% (0.4)	3.9% (0.5)	3.8% (0.5)	4.8% (0.8)	4.4% (0.7)	4.6% (0.9)	4.6% (0.9)	2.9% (0.4)	2.7% (0.4)	<0.001	0.085	0.388	<0.001	1.000	0.060
Margarine	65.8% (3.4)	0.0% (0.0)	68.6% (3.2)	0.0% (0.0)	68.3% (6.7)	0.0% (0.0)	64.1% (7.1)	0.0% (0.0)	75.4% (7.9)	0.0% (0.0)	69.8% (6.2)	0.0% (0.0)	<0.001	<0.001	<0.001	<0.001	<0.001	<0.001
Oils	0.5% (0.3)	100.0% (0.0)	0.9% (0.7)	100.0% (0.0)	5.2% (2.9)	100.0% (0.0)	1.9% (1.9)	100.0% (0.0)	2.8% (2.8)	100.0% (0.0)	1.1% (1.1)	100.0% (0.0)	<0.001	<0.001	<0.001	<0.001	<0.001	<0.001
Discretionary fats	33.6% (3.4)	0.0% (0.0)	30.5% (3.2)	0.0% (0.0)	26.6% (6.2)	0.0% (0.0)	34.0% (7.0)	0.0% (0.0)	21.8% (7.5)	0.0% (0.0)	29.1% (6.2)	0.0% (0.0)	<0.001	<0.001	<0.001	<0.001	0.013	<0.001
**Processed**	2.8% (0.4)	2.9% (0.2)	3.8% (0.5)	2.2% (0.2)	4.3% (0.8)	2.5% (0.3)	5.2% (1.1)	3.9% (1.0)	3.8% (1.1)	2.2% (0.4)	5.6% (0.7)	2.7% (0.3)	0.995	0.002	0.087	0.232	0.245	<0.001
Fruit juices	49.5% (5.3)	100.0% (0.0)	34.6% (4.7)	100.0% (0.0)	36.8% (7.5)	100.0% (0.0)	46.3% (8.4)	100.0% (0.0)	18.2% (9.3)	100.0% (0.0)	48.0% (5.8)	100.0% (0.0)	0.003	<0.001	<0.001	0.034	<0.001	<0.001
Processed fruit	4.2% (2.2)	0.0% (0.0)	0.7% (0.6)	0.0% (0.0)	2.7% (2.1)	0.0% (0.0)	2.8% (2.8)	0.0% (0.0)	0.0% (0.0)	0.0% (0.0)	3.8% (1.8)	0.0% (0.0)	<0.001	0.263	0.373	0.156	1.000	0.439
Cheese	11.4% (3.5)	0.0% (0.0)	11.4% (3.0)	0.0% (0.0)	24.1% (6.4)	0.0% (0.0)	19.3% (6.5)	0.0% (0.0)	16.8% (9.0)	0.0% (0.0)	24.5% (5.2)	0.0% (0.0)	<0.001	<0.001	<0.001	0.002	0.036	<0.001
Processed fish and meat alternatives	8.3% (2.8)	0.0% (0.0)	18.3% (3.9)	0.0% (0.0)	21.9% (6.2)	0.0% (0.0)	4.1% (2.5)	0.0% (0.0)	20.8% (9.3)	0.0% (0.0)	9.7% (2.5)	0.0% (0.0)	0.748	0.002	<0.001	0.906	0.470	0.024
Processed meats	26.7% (4.8)	0.0% (0.0)	35.0% (4.5)	0.0% (0.0)	14.6% (5.3)	0.0% (0.0)	27.5% (7.5)	0.0% (0.0)	44.1% (10.6)	0.0% (0.0)	14.0% (3.8)	0.0% (0.0)	0.007	<0.001	0.324	0.087	<0.001	0.195
**Ultra-processed**	63.7% (1.2)	26.2% (1.1)	55.1% (1.2)	27.4% (1.1)	60.5% (2.2)	27.0% (2.1)	61.0% (2.2)	26.0% (1.9)	56.7% (2.7)	21.8% (2.6)	58.3% (1.7)	26.7% (1.6)	<0.001	<0.001	<0.001	<0.001	<0.001	<0.001
Vegetable dishes	2.6% (0.4)	18.3% (1.6)	2.0% (0.4)	14.7% (1.6)	4.5% (1.1)	19.6% (3.1)	3.5% (1.0)	14.7% (2.3)	0.7% (0.5)	8.1% (2.8)	4.7% (0.9)	20.9% (2.5)	<0.001	<0.001	<0.001	<0.001	0.071	<0.001
Ultra-processed fruit	0.3% (0.1)	0.0% (0.0)	0.1% (0.0)	0.0% (0.0)	1.0% (0.5)	0.0% (0.0)	0.5% (0.3)	0.0% (0.0)	1.5% (0.7)	0.0% (0.0)	0.3% (0.1)	0.0% (0.0)	0.233	0.544	0.003	0.081	0.008	0.271
Cereal, fortified, sugars 15–30 g/100 g	0.4% (0.2)	16.7% (1.7)	0.9% (0.4)	17.2% (1.7)	0.8% (0.4)	19.7% (3.6)	1.0% (0.6)	19.0% (3.4)	1.7% (1.2)	20.6% (4.4)	1.0% (0.5)	16.9% (2.8)	<0.001	<0.001	<0.001	<0.001	<0.001	<0.001
Cereal, unfortified, sugars <15 g/100 g	0.1% (0.1)	13.4% (1.6)	0.0% (0.0)	12.3% (1.5)	0.3% (0.2)	10.8% (2.7)	0.1% (0.1)	16.2% (3.0)	0.3% (0.3)	19.7% (4.6)	0.1% (0.1)	21.5% (3.2)	<0.001	<0.001	<0.001	<0.001	<0.001	<0.001
Wholegrain bread, fortified	2.7% (0.7)	51.6% (2.3)	2.8% (0.6)	55.8% (2.3)	3.8% (0.8)	49.9% (4.3)	4.3% (1.3)	50.1% (4.0)	4.8% (1.8)	51.7% (5.3)	5.6% (1.1)	40.7% (3.3)	<0.001	<0.001	<0.001	<0.001	<0.001	<0.001
Cereal, fortified, sugars <15 g/100 g	6.5% (0.7)	0.0% (0.0)	6.5% (0.8)	0.0% (0.0)	4.8% (1.2)	0.0% (0.0)	7.9% (1.2)	0.0% (0.0)	7.3% (2.2)	0.0% (0.0)	9.2% (1.6)	0.0% (0.0)	<0.001	<0.001	<0.001	<0.001	0.001	<0.001
Wholegrain bread, unfortified	0.6% (0.2)	0.0% (0.0)	1.5% (0.5)	0.0% (0.0)	0.4% (0.3)	0.0% (0.0)	0.3% (0.3)	0.0% (0.0)	0.0% (0.0)	0.0% (0.0)	0.5% (0.2)	0.0% (0.0)	0.132	<0.001	0.616	0.707	1.000	0.413
Refined bread	13.8% (1.2)	0.0% (0.0)	17.6% (1.3)	0.0% (0.0)	10.7% (2.0)	0.0% (0.0)	11.5% (1.8)	0.0% (0.0)	11.9% (2.7)	0.0% (0.0)	8.6% (1.4)	0.0% (0.0)	<0.001	<0.001	<0.001	<0.001	<0.001	<0.001
Cereal, unfortified, sugars >15–30 g/100 g	0.2% (0.1)	0.0% (0.0)	0.2% (0.2)	0.0% (0.0)	0.7% (0.4)	0.0% (0.0)	0.0% (0.0)	0.0% (0.0)	0.3% (0.3)	0.0% (0.0)	0.9% (0.4)	0.0% (0.0)	0.317	0.402	0.095	1.000	0.656	0.043
Batter-based products	0.6% (0.2)	0.0% (0.0)	1.2% (0.3)	0.0% (0.0)	0.7% (0.5)	0.0% (0.0)	0.2% (0.1)	0.0% (0.0)	1.2% (0.9)	0.0% (0.0)	0.7% (0.3)	0.0% (0.0)	0.051	<0.001	0.231	0.795	0.073	0.094
Pasta, with additions	2.0% (0.4)	0.0% (0.0)	0.9% (0.3)	0.0% (0.0)	1.6% (0.6)	0.0% (0.0)	2.0% (1.1)	0.0% (0.0)	2.7% (1.4)	0.0% (0.0)	2.2% (0.6)	0.0% (0.0)	<0.001	0.133	0.068	0.100	0.038	<0.001
Liquid/fortified breakfast	0.3% (0.1)	0.0% (0.0)	0.5% (0.3)	0.0% (0.0)	0.4% (0.2)	0.0% (0.0)	0.6% (0.2)	0.0% (0.0)	0.7% (0.4)	0.0% (0.0)	0.6% (0.2)	0.0% (0.0)	0.080	0.003	0.324	0.125	0.132	0.095
Yoghurt reduced fat	0.4% (0.1)	0.0% (0.0)	0.3% (0.2)	0.0% (0.0)	0.5% (0.2)	0.0% (0.0)	0.4% (0.2)	0.0% (0.0)	0.6% (0.5)	0.0% (0.0)	0.7% (0.2)	0.0% (0.0)	0.040	0.084	0.186	0.628	0.094	0.004
Flavored milk	0.5% (0.2)	0.0% (0.0)	0.0% (0.0)	0.0% (0.0)	0.3% (0.2)	0.0% (0.0)	0.4% (0.3)	0.0% (0.0)	0.0% (0.0)	0.0% (0.0)	0.5% (0.2)	0.0% (0.0)	<0.001	0.841	0.590	0.100	1.000	0.007
Dairy milk substitutes	0.0% (0.0)	0.0% (0.0)	0.0% (0.0)	0.0% (0.0)	0.2% (0.1)	0.0% (0.0)	0.0% (0.0)	0.0% (0.0)	0.4% (0.3)	0.0% (0.0)	0.5% (0.4)	0.0% (0.0)	0.953	1.000	0.808	1.000	0.297	0.038
Cheese, ultra-processed	0.0% (0.0)	0.0% (0.0)	0.1% (0.0)	0.0% (0.0)	0.0% (0.0)	0.0% (0.0)	0.0% (0.0)	0.0% (0.0)	0.0% (0.0)	0.0% (0.0)	0.0% (0.0)	0.0% (0.0)	0.599	0.037	1.000	1.000	1.000	0.448
Discretionary	69.0% (1.5)	0.0% (0.0)	65.6% (1.6)	0.0% (0.0)	69.5% (2.6)	0.0% (0.0)	67.3% (2.3)	0.0% (0.0)	66.1% (4.0)	0.0% (0.0)	63.7% (2.3)	0.0% (0.0)	<0.001	<0.001	<0.001	<0.001	<0.001	<0.001

^1^ All data are mean (SEM) percentage of all diets used for analysis. ^2^ For statistical significance, *p* < 0.005. A hyphen indicates that the values for comparison were exactly the same among all samples from within a group, and thus between both current vs. healthier diets, and low vs medium socioeconomic households. Food categories were defined as follows: ‘Discretionary fats’, included butter and cream; ‘Processed fruit’, included commercial/canned fruit in juice; ‘Processed fish and meat alternatives’, included canned fish, canned legumes, tofu, and other processed meat alternatives; ‘Vegetable dishes’, included canned vegetable soups, commercially produced coleslaws, potato salad and frozen vegetables and ‘heat and eat’ vegetable dishes; ‘Ultra-processed fruit’, included commercial/canned fruit with added sugars; ‘Refined bread’, included commercial white breads both unfortified, and fortified with iodine, or iodine and folic acid; ‘Wholegrain bread, fortified’, included commercial whole meal/wholegrain breads, fortified with iodine, or iodine and folic acid; ‘Dairy milk substitutes’, included commercially-produced almond, rice and soy milk. C, current diet; H, healthier diet; M, Maori household; P, pacific household; N, NZEO household; NZEO, New Zealand European and other ethnic groups; SES, socioeconomic status.

## Data Availability

Information about and access to publicly available NZ nutrition survey data for both adults and children can be sourced here: https://www.health.govt.nz/nz-health-statistics/national-collections-and-surveys/surveys/past-surveys/nutrition-survey (accessed on 25 October 2020) [6,25]; access to the NZ Food Composition Database can be found here: https://www.foodcomposition.co.nz/foodfiles/ (accessed on 25 October 2020) [21]; access to the 2020 NRAUS Australia New Zealand Food Category Cost Dataset can be found here: https://datadryad.org/stash/dataset/doi:10.5061/dryad.gb5mkkwq0 [23].

## Supplementary Materials

The following are available online at https://www.mdpi.com/article/10.3390/ijerph18157950/s1. Methods S1: The full calculation of NRF9.3, adapted for the NZ Nutrient Reference Values; Methods S2: Substitution modeling protocol rules; Table S1: Details and descriptions of all 61 food categories; Table S2: Nutrient density and NOVA processing level of all 61 food categories; Table S3: Nutrient reference values for each household; Table S4: Micronutrient analysis of current and healthier, low-cost diets; Table S5: Individual component scores for the HEIFA-2013 and DICE.
