# Supplementary material for: Nutrient Dense, Low-Cost Foods Can Improve the Affordability and Quality of the New Zealand Diet—A Substitution Modeling Study
Source: Int J Environ Res Public Health. 2021 Jul 27;18(15):7950. doi: 10.3390/ijerph18157950 (PMC8345759; doi:10.3390/ijerph18157950)
Supplement: Supplementary file 1 [file ijerph-18-07950-s001.zip › Kellogg_Cereal Economics_SUPPLEMENTARY METHODS S1_IJERPH_NZ_20210709.pdf]

Supplementary Methods S1. The calculation of NRF9.3, adapted to the New Zealand Dietary Guidelines and Nutrient Reference Values.

The NRF9.3/100 kcal provides an algorithm to assess the proportion of nutrient requirements that are provided by a food, in relation to the energy provided by the food. There are nine positively weighted nutrients (protein, fibre, calcium, iron, potassium, magnesium, and vitamins A, C, and E), and three negatively weighted nutrients (saturated fat, added sugar, and sodium).

The NRF9.3/100 kcal was calculated for each of the three representative foods in the 61 food categories (189 foods in total), with daily values calculated as a percentage per 100 kcal and capped at 100%.

The complete calculation for the NRF9.3/100 kcal is:

1. NR9 subscore:

$$\text{NR9/serving} = \sum_{1\text{–}9} (\text{nutrient/daily value}) \times 100$$

$$\text{NR9/100 kcal} = \sum_{1\text{–}9} (\text{nutrient/daily value})/(\text{kcal per serve}) \times 100$$

2. LIM subscore:

$$\text{LIM/serving} = \sum_{1\text{–}3} (\text{nutrient/maximum recommended value}) \times 100$$

$$\text{LIM/100 kcal} = \sum_{1\text{–}3} (\text{nutrient/maximum recommended value})/(\text{kcal per serve}) \times 100$$

3. Composite NRF9.3/100 kcal

$$\text{NRF9.3/100 kcal} = \text{NR9}_{100\text{ kcal}} - \text{LIM}_{100\text{ kcal}}$$

Since the nutrients used in the NRF9.3 are mostly in line with the Nutrient Profiling Scoring Criterion (NPSC, Food Standards Australia and New Zealand/FZANZ), it was not necessary to change all the nutrients included in the NRF 9.3, modified for New Zealand [31]. However, the full set of reference values were determined based on the following additional criteria:

a. The reference used was an adult aged 18-50 years.

b. When males and females had a different recommended dietary intake (RDI) or adequate intake (AI), the higher value was chosen. For example, the RDI for iron for males was 8 mg/day, and for females was 18 mg/day; therefore, the higher value of 18 mg/day was chosen.

c. When males and females had different maximum recommended values (MRV), the lower value was chosen. For example, the saturated fat MRV is 9% of dietary energy. The total dietary energy was based on the lowest average daily energy requirement for a sedentary working female (physical activity level [PAL] of 1.6) [31].

d. The upper limit for Added Sugar was based on the WHO recommendations that added sugar should not be more than 10% of dietary energy, assuming an energy intake of 8400 kJ/day for an adult female, as per the rationale above [31].

e. The upper limit for Sodium was based on suggested dietary target to reduce chronic disease in the NRVs (<2,000 mg/day sodium) [31].

Table: Reference Values from NRF 9.3 [12] and corresponding Australian reference values [31] used in the current study.

	RDV (US)	MRV (US)	RDI/AI (NZ)	MRV (NZ)
Protein (g)	50		64	
Fibre (g)	25		30	
Vitamin A	5000 IU		900 µg/day	
Vitamin C (mg)	60		45	
Vitamin E (mg)	20		10	
Calcium (mg)	1000		1000	
Iron (mg)	18		18	
Potassium (mg)	3500		3800	
Magnesium (mg)	400		420	
Saturated fat (g)		20		20
Added Sugar (g)		50		50
Sodium (mg)		2400		2000

RDV: Reference daily value. MRV: Maximum recommended value. US: United States. RDI: Recommended dietary intake. AI: Adequate intake. NZ: New Zealand.
