# Supplementary material for: Nutrient Dense, Low-Cost Foods Can Improve the Affordability and Quality of the New Zealand Diet—A Substitution Modeling Study
Source: Int J Environ Res Public Health. 2021 Jul 27;18(15):7950. doi: 10.3390/ijerph18157950 (PMC8345759; doi:10.3390/ijerph18157950)
Supplement: Supplementary file 1 [file ijerph-18-07950-s001.zip › Kellogg_Cereal Economics_SUPPLEMENTARY METHODS S2_IJERPH_NZ_20210709.pdf]

Supplementary Methods S2: Rules used to develop the substitution modelling protocol.

The substitution modelling algorithm was developed and employed according to the following substitution rules:

1. All substituted healthier diets aim to meet New Zealand Dietary Guidelines for core food groups [34], with no allowance for discretionary foods.

2. Number of servings for each core food group was calculated by dividing the weight of each food category consumed by the weight per serve, as defined in the New Zealand Dietary Guidelines [34,39], followed by summing the serves of all food categories within a core food group. For example, for the vegetables core food group, the number of servings was totalled from the following food categories: potatoes, unprocessed; orange/yellow vegetables; green leafy vegetables; other vegetables; vegetable dishes. One discretionary serving was calculated as being equivalent to 150 kcal.

3. All substitutions replaced food categories in the current diet that placed in the lowest three nutrient density-to-cost ratio quartiles with core food categories from the highest quartile of nutrient density to cost [15], as identified in Step 1 of the modelling protocol.

4. Where more than one replacement option in the highest quartile was possible, selection of the replacement food category was random. For example, green leafy vegetables (quartile 2) could be replaced with orange/yellow vegetables (quartile 1); potatoes and red kumara, unprocessed (quartile 1); vegetable dishes (quartile 1); or other vegetables (quartile 1).

5. If no food category was appropriate to be substituted using the highest quartile of nutrient density to cost, the highest-ranking food category from the second quartile was used. For example, there were no dairy products in the top quartile, so the highest-ranking dairy products/dairy alternatives for each food type, within the 2nd quartile, were used. If there were no 2nd quartile options within a food type, the top 3rd quartile option within the relevant food group was made.

6. Each substitution was made according to the 'like for like' principle, whereby each food category in the current diet was substituted for a healthier (more nutrient-dense) version of that food, keeping as close to the original food as possible. For example, ready-to-eat breakfast cereal with sugars >30g/100g (discretionary) would be substituted with ready-to-eat breakfast cereal, unfortified with sugars <15g/100g (quartile 1) or ready-to-eat breakfast cereal, fortified with sugars 15-30g/100g (quartile 1); while flavoured milk (quartile 3) would be substituted for skim milk.

7. The substitution of discretionary food categories for core food categories was also made according to the 'like for like' principle. For example, butter were replaced with plant based oils. Where a direct substitution was not possible due to there being no similar core food, the selection of the replacement food was based upon cost and the judgment of the investigators. For example, bakery desserts were replaced with a random selection from the top quartile of food categories from the grains and cereals core food group.

8. If a ‘like for like’ substitution was not possible due to the dietary guidelines for that food category already being met; or the food category being discretionary and needing to be replaced with a core food category, the substitution was made using a more nutrient-dense, lower cost food category from the next most lacking core food group, according to the following hierarchy, based on the highest to the lowest level of intake within the current New Zealand diet across both children and adults (4,27): fruit > vegetables > grains > dairy and alternatives > meat and alternatives. The exception was sauces, which, due to being a discretionary food used in the main meal, were explicitly replaced with food categories from the vegetables food group.

9. In cases when a healthier version of the food to be substituted was not available, the selection of the replacement food was based upon cost and the judgment of the investigators.
