# Supplementary material for: Nutrient Dense, Low-Cost Foods Can Improve the Affordability and Quality of the New Zealand Diet—A Substitution Modeling Study
Source: Int J Environ Res Public Health. 2021 Jul 27;18(15):7950. doi: 10.3390/ijerph18157950 (PMC8345759; doi:10.3390/ijerph18157950)
Supplement: Supplementary file 1 [file ijerph-18-07950-s001.zip › Kellogg_Cereal Economics_SUPPLEMENTARY TABLE S1_IJERPH_NZ_20210709.pdf]

**Supplementary Table S1.** Details and descriptions of all 61 food categories, including the Food Chapter each food category belongs to, according to the New Zealand FOODFiles Food Composition Database [19].

| **Food chapter**[1] | | **Food category** | |
|---|---|---|---|
| | | **Name** | **Description** |
| A | Bakery products | Breads and rolls, wholegrain[2], fortified[3] | Bagels, bread, English muffins, and flat breads with additional fortification, all grains including gluten-free varieties |
| | | Breads and rolls, refined grain, fortified | White (including gluten-free) bagels, bread, English muffins, and flat breads with additional fortification |
| | | Breads and rolls, wholegrain, unfortified | Bagels, bread, English muffins, and flat breads with no fortification, all grains including gluten-free varieties |
| | | Breads and rolls, refined grain, unfortified | White (including gluten-free) bagels, bread, English muffins, and flat breads with no fortification |
| | | Bakery desserts | Sweet buns, sweet biscuits, cakes, cupcakes, banana breads, sweet scones and muffins, pudding, pie, danishes, éclairs, doughnuts, pavlova |
| | | Savoury biscuits | Crackers, wafers, rice cakes, and crispbreads |
| C | Beverages, non-alcoholic | Fruit juices | Commercial and non-commercial juices |
| | | Sweetened beverages | Fruit drinks, cordials, soft drinks, flavoured mineral waters, sports drinks, and energy drinks |
| | | Liquid/fortified breakfast | Milo prepared with milk, unfortified beverage flavourings prepared with milk, beverage bases unmixed, and breakfast cereal beverages |
| | | Dairy milk substitutes | Soy beverages and rice beverages including smoothies and milkshakes |
| | | Flavoured milk | Iced coffee, milk with flavouring, milkshakes, thick-shakes, and fruit smoothies with milk |
| D | Breakfast cereals | Breakfast cereal, RTE, fortified, sugar ≤ 15g/100g[4] | Fortified ready-to-eat breakfast cereals and muesli varieties, with or without fruit and nuts, and lower in sugar. *E.g., Weet-bix, cornflakes, rice bubbles* |
| | | Breakfast cereal, RTE, fortified, sugar > 15g but ≤30g/100g | Fortified ready-to-eat breakfast cereals and muesli, with or without fruit and nuts higher in sugar. *E.g., All Bran, Special K* |

| | | | |
|---|---|---|---|
| | | Breakfast cereal, RTE, unfortified, sugar ≤ 15g/100g | Unfortified ready-to-eat breakfast cereals and muesli varieties, lower in sugar. *Eg, Puffed wheat, unfortified wheat biscuits* |
| | | Breakfast cereal, RTE unfortified, sugar > 15g but ≤30g/100g | Unfortified ready-to-eat breakfast cereals and muesli higher in sugar. *Eg, muesli, unfortified breakfast flakes with dried fruit added* |
| | | Breakfast cereal, RTE, sugar > 30g/100g | Discretionary ready-to-eat breakfast cereals E*g, Cocoa Pops* |
| | | Hot porridge | Porridge with and without (milk, sugar, honey and dried fruit) |
| E | Cereals and pseudo-cereals | Pasta and noodles | Pasta and noodles, plain without additions |
| | | Pasta and noodles, with additions | Pasta and noodles with additions, such as canned spaghetti in tomato sauce, ravioli, and macaroni cheese |
| | | Rice and grains | Rice, quinoa and couscous without additions |
| F | Dairy | Dairy milk full-fat | Fluid milk, condensed milk, and milk powder full-fat (3.3%) |
| | | Dairy milk reduced fat | Fluid milk, condensed milk, and milk powder, fat 1.5% |
| | | Dairy milk skim | Fluid milk, condensed milk, and milk powder, fat <1% |
| | | Yoghurt full-fat | Yoghurt, plain and sweetened varieties, > 3% fat |
| | | Yoghurt reduced fat | Yoghurt, plain and sweetened varieties, ≤ 3% fat |
| | | Cream | Cream, sour cream, and whipped cream |
| | | Cheese | Cheese blocks and grated cheese, all varieties |
| | | Cheese ultra-processed[5] | Cheese slices ('plastic cheese') |
| | | Ice-cream and ice-confections | Ice-cream, sweetened ice desserts, gelato |
| | | Butter | Butter |

| | | | |
|---|---|---|---|
| G | Eggs | Eggs | Eggs, without additions |
| J | Fats and oils | Oils | Vegetable and seed oils |
| | | Margarine and margarine-like spreads | Dairy blends, margarines and table spreads |
| K | Fin fishes | Fresh fish | Fish (baked, boiled, roasted, smoked) |
| | | Fish, battered and fried | Fish (battered/crumbed and fried, including frozen) |
| | | Canned fish | Canned tuna, salmon, sardines, mackerel |
| L | Fruits | Fresh fruit | Raw fruits and fruit salads |
| | | Commercial fruit, no added sugar | Canned fruits (drained, with juice) |
| | | Commercial fruit with added sugar | Canned fruits (with syrup) |
| | | Dried fruits | Dried fruits |
| M | Meats | Red meat, fat fully trimmed | Beef, lamb, pork, veal, kangaroo with all fat removed |
| | | Red meat, fat semi or untrimmed | Beef, lamb, pork, veal, kangaroo with fat still present, or only partially removed |
| | | Poultry | Chicken and duck |
| N | Meat products | Ultra-processed meats | Bacon, ham, prosciutto, salami, corned beef, turkey deli-sliced, spam, and beef jerky |
| | | Processed meats | Sausages (beef, pork, chicken), frankfurters, saveloys |
| Q | Nuts and seeds | Nuts and seeds | Chia seeds, nuts (peanuts, almonds, pistachio, pine), nut butters |
| R | Recipes[6] | Batter-based products | Crepes, pancakes, pikelet, waffles, fritters |

| | | | |
|---|---|---|---|
| | | Dips | Dairy-based dips, guacamole, and hummus |
| S | Sauces | Sauces, cream-based | Cream/oil based sauces used frequently in the NZ diet, such as coconut cream, cream-based simmer sauces, and mayonnaise |
| | | Sauces, tomato-based | Tomato-based sauces used frequently in the NZ diet, such as tomato sauce, canned tomatoes and tomato-based pasta sauce |
| T | Shellfishes | Shellfish | Crabs, prawns, scallops, squid |
| U | Savoury snack foods | Savoury snacks | Potato crisps, corn chips, popcorn, snack mixes, and 2-minute noodles |
| W | Sugars, confectionaries, sweet spreads | Sweet spreads and syrups | Sugar, honey, and maple syrups, jams and sweet spreads |
| | | Sweet snacks | Chocolate, bars, licorice, honeycomb, lollies, marshmallows, muesli and cereal bars |
| X | Vegetables and pulses | Potatoes, red kumara, and taro unprocessed | Baked, boiled, fried, grilled, or BBQ'd potatoes, Owairaka Red kumara and taro. |
| | | Potatoes, red kumara and taro, processed | Fries, hash-browns, and wedges (potatoes, Owairaka Red kumara and taro). |
| | | Orange/yellow vegetables | Carrots, orange/gold kumara, pumpkin |
| | | Green leafy vegetables | Lettuce, spinach, silverbeet, bok-choy, watercress |
| | | Other vegetables | Broccoli, cabbage and cauliflower, leeks, onions, peas and beans, beetroot, root vegetables that are not orange or yellow, zucchini, tomato, mushrooms, onions, leeks, and green bananas |
| | | Vegetable dishes | Vegetable casseroles, vegetable curries, vegetable stir-fry, vegetable salads without additions, coleslaw, and potato salad |
| | | Meat substitutes/legumes and dishes | Beans, lentils, baked beans plain and in (sauces, curry, salads and casseroles), tofu, and tempeh |

[1] Each food category can be matched to one of the food chapters outlined in the New Zealand Food Composition Database (25).
[2] Where specified, wholegrain versions of a food were grouped together; thus, refined versions of a food were also grouped together.
[3] Where specified, fortified foods were grouped together; non-fortified foods were thus also grouped together.

[4] Where applicable, classifications based on nutritional properties were used: High or moderate fat foods were grouped together, and reduced or skimmed versions of a food were grouped together; Foods with a similar sugar content were grouped together; Foods high in sugar and classified as discretionary were grouped together.
[5] Where possible, foods within each level of processing (NOVA classification) were grouped together.
[6] A selection of foods were excluded. These were: mixed dishes and takeaway foods, due to them containing more than one other food category and therefore creating added complexity that would be unfeasible to model; organ meats and offal, due to a low frequency of consumption; tea and coffee, due to contributing negligible calories to the diet.; water, due to contributing negligible calories to the diet; alcohol, due to contributing negligible nutrients to the diet; supplements, due to contributing negligible calories to the diet; infant formula and baby foods, due to no household member being an infant or baby.
