# Supplementary material for: Nutrient Dense, Low-Cost Foods Can Improve the Affordability and Quality of the New Zealand Diet—A Substitution Modeling Study
Source: Int J Environ Res Public Health. 2021 Jul 27;18(15):7950. doi: 10.3390/ijerph18157950 (PMC8345759; doi:10.3390/ijerph18157950)
Supplement: Supplementary file 1 [file ijerph-18-07950-s001.zip › Kellogg_Cereal Economics_SUPPLEMENTARY TABLE S2_IJERPH_NZ_20210709.pdf]

Supplementary Table S2. Nutrient density (NRF9.3/100 kcal), cost (NZD/100 kcal) and the nutrient density to cost ratio (with quartile rankings) for all 61 food categories. The classification according to the NOVA processing levels is also shown.

Food categories[1]	NRF9.3/100 kcal	$/100 kcal	Nutrient density to cost ratio[2]	Quartile	Processing Level (NOVA)
**Vegetables**					
Other vegetables	355.69 (162.8)	1.31 (0.8)	337.86 (179.9)	1	Unprocessed
Orange/yellow vegetables	205.28 (88.1)	0.93 (0.3)	324.29 (335.0)	1	Unprocessed
Potatoes, red kumara, and taro, unprocessed	96.38 (19.9)	0.57 (0.3)	196.05 (77.7)	1	Unprocessed
Vegetable dishes	99.74 (90.8)	0.64 (0.4)	183.22 (192.9)	1	Ultra-processed
Green leafy vegetables	428.45 (181.2)	7.10 (4.8)	72.06 (30.8)	2	Unprocessed
**Fruit**					
Fruit juices	272.15 (105.8)	0.71 (0.3)	415.97 (102.7)	1	Processed
Fresh fruit	116.57 (106.8)	0.72 (0.4)	159.12 (84.2)	1	Unprocessed
Dried fruit	24.25 (4.2)	0.21 (0.1)	126.80 (46.0)	1	Unprocessed
Commercial fruit, no added sugar	88.93 (46.4)	0.76 (0.1)	122.07 (78.2)	2	Processed
Commercial fruit with added sugar	48.85 (33.2)	0.47 (0.1)	103.02 (62.6)	2	Ultra-processed
**Cereals and grain foods**					
Hot porridge	27.51 (9.8)	0.18 (0.1)	245.98 (153.6)	1	Unprocessed
Pasta and noodles	14.16 (6.8)	0.19 (0.2)	180.91 (137.1)	1	Unprocessed
Breads and rolls, wholegrain, fortified	19.43 (2.2)	0.14 (0.0)	150.51 (55.1)	1	Ultra-processed
Breakfast cereal, RTE, unfortified, sugar <15g/100g	23.14 (0.0)	0.16 (0.0)	148.88 (3.5)	1	Ultra-processed
Breakfast cereal, RTE, fortified, sugar 15-30g/100g	46.41 (19.8)	0.31 (0.0)	148.35 (58.4)	1	Ultra-processed
Rice and grains	10.53 (6.3)	0.08 (0.0)	136.35 (70.3)	1	Unprocessed
Breakfast cereal, RTE, fortified, sugar <15g/100g	23.36 (21.7)	0.18 (0.0)	121.30 (100.6)	2	Ultra-processed
Breads and rolls, wholegrain, unfortified	19.97 (4.9)	0.27 (0.1)	80.63 (31.6)	2	Ultra-processed
Breads and rolls, refined grain, fortified	7.35 (0.0)	0.13 (0.0)	60.31 (20.0)	3	Ultra-processed
Breakfast cereal, RTE unfortified, sugar 15-30g/100g	10.80 (5.7)	0.19 (0.0)	58.03 (33.8)	3	Ultra-processed

Breads and rolls, refined grain, unfortified	12.01 (0.4)	0.27 (0.1)	46.51 (12.9)	3	Ultra-processed
Pasta and noodles, with additions	10.62 (14.0)	0.68 (0.3)	15.64 (13.4)	4	Ultra-processed
Batter-based products	4.54 (9.1)	0.68 (0.2)	6.60 (9.9)	4	Ultra-processed
**Milk, yoghurt, cheese and alternatives**					
Dairy milk skim	68.93 (6.3)	0.56 (0.1)	124.52 (15.6)	2	Unprocessed
Dairy milk reduced fat	46.55 (0.0)	0.44 (0.0)	107.85 (12.1)	2	Unprocessed
Liquid/fortified breakfast	38.47 (50.7)	0.59 (0.3)	90.48 (96.9)	2	Ultra-processed
Dairy milk full-fat	28.47 (0.0)	0.34 (0.0)	85.23 (9.6)	2	Unprocessed
Dairy milk substitutes	60.16 (76.9)	0.95 (0.3)	59.74 (57.7)	3	Ultra-processed
Cheese	12.90 (4.4)	0.28 (0.0)	45.97 (10.3)	3	Processed
Yoghurt full-fat	27.26 (10.2)	0.63 (0.2)	42.52 (10.2)	3	Unprocessed
Yoghurt reduced fat	33.89 (30.9)	0.69 (0.2)	42.06 (26.7)	3	Ultra-processed
Flavoured milk	9.97 (9.1)	0.37 (0.1)	23.46 (21.9)	3	Ultra-processed
Cheese ultra-processed	-0.35 (3.5)	0.45 (0.2)	1.81 (4.2)	4	Ultra-processed
**Meat and alternatives**					
Nuts and seeds	35.45 (11.8)	0.11 (0.0)	303.61 (50.7)	1	Unprocessed
Eggs	50.91 (0.0)	0.43 (0.1)	125.93 (26.8)	2	Unprocessed
Meat substitutes/legumes and dishes	46.54 (15.6)	0.61 (0.6)	123.14 (65.2)	2	Unprocessed
Poultry	26.63 (13.3)	0.45 (0.3)	64.79 (24.2)	2	Unprocessed
Canned fish	34.75 (5.3)	0.87 (0.5)	56.34 (36.5)	3	Processed
Shellfish	64.64 (16.3)	1.66 (0.5)	40.53 (8.2)	3	Unprocessed
Fresh fish	41.03 (17.5)	2.38 (1.4)	30.82 (32.4)	3	Unprocessed
Red meat, fat fully trimmed	32.82 (11.8)	1.56 (0.4)	22.40 (10.5)	4	Unprocessed
Red meat, fat semi or untrimmed	-6.34 (18.3)	0.73 (0.2)	7.61 (8.9)	4	Unprocessed
**Other foods**					
Oils	2.87 (4.8)	0.04 (0.0)	138.54 (103.9)	1	Culinary
Margarine and margarine-like spreads	9.12 (9.6)	0.08 (0.0)	102.83 (63.0)	2	Culinary
**Discretionary foods**					

Potatoes, red kumara, and taro, processed	25.39 (10.3)	0.24 (0.1)	129.20 (81.4)	1	Ultra-processed
Sauces, tomato-based	64.16 (43.1)	0.70 (0.4)	98.70 (35.0)	2	Ultra-processed
Breakfast cereal, RTE, sugar >30g/100g	20.40 (10.3)	0.36 (0.1)	80.58 (74.0)	2	Ultra-processed
Sweetened beverages	14.71 (53.5)	2.47 (3.1)	49.46 (74.6)	3	Ultra-processed
Sweet snacks	4.98 (22.6)	0.28 (0.0)	44.35 (34.6)	3	Ultra-processed
Dips	24.24 (23.5)	0.70 (0.3)	38.90 (32.3)	3	Ultra-processed
Fish, battered and fried	21.17 (0.0)	0.74 (0.1)	29.27 (4.0)	3	Ultra-processed
Savoury snacks	0.66 (4.9)	0.19 (0.1)	11.75 (12.6)	4	Ultra-processed
Savoury biscuits	0.51 (6.2)	0.29 (0.1)	7.82 (8.6)	4	Ultra-processed
Butter	0.56 (0.9)	0.14 (0.0)	3.95 (5.8)	4	Culinary
Cream	-9.23 (2.0)	0.32 (0.2)	0.00 (0.0)	4	Culinary
Ultra-processed meats	-10.87 (6.7)	0.53 (0.2)	0.00 (0.0)	4	Ultra-processed
Ice-cream and ice-confections	-11.73 (7.0)	0.14 (0.1)	0.00 (0.0)	4	Ultra-processed
Sauces, cream-based	-12.15 (7.2)	0.27 (0.1)	0.00 (0.0)	4	Ultra-processed
Bakery desserts	-15.26 (13.3)	0.25 (0.1)	0.00 (0.0)	4	Ultra-processed
Sweet spreads and syrups	-18.40 (12.5)	0.24 (0.1)	0.00 (0.0)	4	Ultra-processed
Processed meats	-20.28 (6.4)	0.58 (0.2)	0.00 (0.0)	4	Processed

[1] All data is presented as mean (SEM).

[2] The nutrient density to cost ratio was calculated according to the following formula: (mean NRF9.3/100 kcal) / (mean cost/100 kcal). The nutrient density-to-cost ratio for a food category is the mean of the ratios of the representative foods within each food category.
