# Supplementary material for: Nutrient Dense, Low-Cost Foods Can Improve the Affordability and Quality of the New Zealand Diet—A Substitution Modeling Study
Source: Int J Environ Res Public Health. 2021 Jul 27;18(15):7950. doi: 10.3390/ijerph18157950 (PMC8345759; doi:10.3390/ijerph18157950)
Supplement: Supplementary file 1 [file ijerph-18-07950-s001.zip › Kellogg_Cereal Economics_SUPPLEMENTARY TABLE S3_IJERPH_NZ_20210709.pdf]

Supplementary Table S3. Recommended core food group intakes and selected nutrient reference values for each household member and total household.

Required Food Group/Nutrient	Household member				Total Household[3]
	Male 31-50	Female 31-50	Male 11-14[2]	Female 7-10[2]	
**Core Food Groups (servings)[1]**					
Vegetables	6	5	5.5	5	21.5
Fruit	2	2	2	2	8
Grains	6	6	6	4	22
Lean meat and alternatives	3	2.5	2.5	2	10
Milk and milk products	2.5	2.5	3	2.5	10.5
**Macronutrients[4]**					
Protein (% energy, AMDR)	15-25	15-25	15-25	15-25	15-25
Total Fat (% energy, MRV)	35	35	35	35	35
Saturated Fat (% energy, MRV)	10	10	10	10	10
Total Carbohydrates (% energy, AMDR)	45-65	45-65	45-65	45-65	45-65
Added Sugar (% energy, MRV)	10	10	10	10	10
Fibre (g/day)	30	25	26	19	100
**Micronutrients[5]**					
Vitamin A (μg/day)	900	700	750	500	2850
Thiamine (mg/day)	1.2	1.1	1.05	0.75	4.1
Riboflavin (mg/day)	1.3	1.1	1.1	0.75	4.25
Niacin (mg/day)	16	14	14	10	54
Vitamin B6 (mg/day)	1.3	1.3	1.15	0.8	4.55
Vitamin B12 (μg/day)	2.4	2.4	2.1	1.5	8.4
Folate (μg/day)	400	400	350	250	1400
Vitamin E (mg/day)	10	7	9.5	7	33.5
Calcium (mg/day)	1000	1000	1300	850	4150
Iron (mg/day)	8	18	10.5	10	46.5
Magnesium (mg/day)	420	320	325	185	1250
Potassium (mg/day)	3800	2800	3300	2400	12300
Sodium (mg/day, MRV)	2300	2300	2150	1700	8450
Zinc (mg/day)	14	8	9.5	5	36.5

[1] Total household recommended core food group intakes and nutrient reference values were calculated as the sum for all household members.
[2] The recommended core food group intakes and nutrient reference values for the age group of each child were taken as the average of the intakes and NRVs specified for all contributing age brackets (as used in the New Zealand dietary guidelines [34, 39] and Nutrient Reference Values for Australia and New Zealand [31] respectively. For example, the recommended intake of the vegetables core food group for a female 7-10 years is the average of the recommended intakes of females aged 4-8 years and 9-13 years.

[3] Macronutrient requirements were taken from the Nutrient Reference Values for Australia and New Zealand (33). The values for protein, total fat, saturated fat, and total carbohydrate, are those to prevent chronic disease. For fibre, the RDI was used. Added sugar is based on the recommendation of the World Health Organisation (WHO) (33).
[4] Micronutrient requirements were taken from the Nutrient Reference Values for Australia and New Zealand (33). The value for sodium is the maximum recommended value to prevent chronic disease. AMDR, acceptable macronutrient distribution range; MRV, maximum recommended value.
