# Supplementary material for: Nutrient Dense, Low-Cost Foods Can Improve the Affordability and Quality of the New Zealand Diet—A Substitution Modeling Study
Source: Int J Environ Res Public Health. 2021 Jul 27;18(15):7950. doi: 10.3390/ijerph18157950 (PMC8345759; doi:10.3390/ijerph18157950)
Supplement: Supplementary file 1 [file ijerph-18-07950-s001.zip › Kellogg_Cereal Economics_SUPPLEMENTARY TABLE S4_IJERPH_NZ_20210709.pdf]

Supplementary Table S4. Micronutrient analysis of the current **(A)** and healthier, low-cost **(B)** diets, for each ethnic population group of both low and medium socioeconomic households.

A.

**Micronutrients (per day)[1,2]: Current diet**	**Low socioeconomic household**			**Medium socioeconomic household**		
	**Maori**	**Pacific**	**NZEO**	**Maori**	**Pacific**	**NZEO**
**n**	391	420	133	116	66	190
Vitamin A (µg) *(2850µg/d)*	1547.6 (40.8)	1366.6 (30.0)	1590.1 (63.7)	1418.7 (60.5)	1409.1 (95.6)	1567.7 (58.1)
Thiamine (mg) *(4.1mg/d)*	6.4 (0.2)	6.6 (0.2)	4.7 (0.2)	6.2 (0.3)	7.0 (0.7)	7.2 (0.3)
Riboflavin (mg) *(4.25mg/d)*	6.9 (0.2)	6.2 (0.1)	5.9 (0.2)	6.4 (0.2)	7.5 (0.4)	6.6 (0.2)
Niacin (mg) *(54mg/d)*	104.9 (2.0)	112.4 (1.8)	82.1 (2.2)	95.0 (3.2)	118.4 (5.9)	93.8 (2.4)
Vitamin B6 (µg) *(4.55µg/d)*	8.8 (0.1)	9.5 (0.1)	6.9 (0.2)	7.8 (0.2)	9.5 (0.4)	7.3 (0.2)
Folate (µg) *(1400µg/d)*	1283.4 (46.6)	1344.7 (29.0)	1037.2 (35.8)	1267.9 (46.7)	1416.4 (104.5)	1369.9 (48.7)
Vitamin B12 (µg) *(8.4µg/d)*	11.0 (0.3)	9.9 (0.2)	7.8 (0.3)	10.1 (0.6)	11.7 (0.7)	8.6 (0.3)
Vitamin E (µg) *(33.5µg/d)*	32.1 (0.6)	29.3 (0.5)	24.8 (0.7)	26.4 (0.7)	27.7 (1.3)	23.3 (0.5)
Calcium(mg) *(4150mg/d)*	2099.4 (42.4)	1878.0 (36.1)	2200.5 (66.3)	2089.8 (79.7)	2038.3 (113.7)	2145.0 (56.2)
Iron (mg) *(46.5mg/d)*	41.8 (1.1)	40.3 (0.9)	32.6 (1.1)	39.6 (1.7)	46.2 (3.2)	41.9 (1.5)
Magnesium (mg) *(1250mg/d)*	993.5 (15.7)	1063.7 (15.7)	901.7 (22.0)	911.4 (23.8)	995.8 (36.4)	920.4 (18.0)
Potassium (mg) *(12300mg/d)*	10009.1 (153.5)	10551.6 (161.6)	8249.4 (175.1)	9115.8 (231.3)	10343.2 (401.1)	8559.4 (172.6)
Sodium (mg) *(8450mg/d)*	8858.8 (150.0)	8522.3 (158.6)	6364.0 (173.7)	7159.2 (216.0)	8532.0 (383.0)	6796.1 (181.6)
Zinc (mg) *(36.5mg/d)*	31.6 (0.7)	30.3 (0.5)	24.8 (0.7)	27.0 (0.9)	31.8 (1.3)	27.9 (0.7)

B.

Micronutrients (per day)[1,2]: Healthier diet	Low socioeconomic household			Medium socioeconomic household			Statistical comparisons (p-value)[3]					
							Current vs healthier diet low SES			Current vs healthier diet medium SES		
	**Maori**	**Pacific**	**NZEO**	**Maori**	**Pacific**	**NZEO**	**Maori**	**Pacific**	**NZEO**	**Maori**	**Pacific**	**NZEO**
n	391	420	133	116	66	190						
Vitamin A (µg) *(2850µg/d)*	1792.8 (60.1)	1711.2 (68.7)	2333.8 (146.4)	2523.4 (270.9)	2389.4 (405.1)	1615.4 (73.6)	0.001	<0.001	<0.001	0.012	0.200	0.639
Thiamine (mg) *(4.1mg/d)*	7.5 (0.2)	7.3 (0.1)	5.6 (0.2)	5.8 (0.2)	6.2 (0.3)	5.8 (0.2)	<0.001	0.001	<0.001	0.308	0.283	<0.001
Riboflavin (mg) *(4.25mg/d)*	8.9 (0.3)	8.1 (0.2)	7.8 (0.3)	7.2 (0.3)	7.3 (0.4)	7.6 (0.3)	<0.001	<0.001	<0.001	0.048	0.687	0.002
Niacin (mg) *(54mg/d)*	117.0 (2.0)	115.5 (1.7)	88.3 (2.0)	94.8 (2.7)	106.4 (3.8)	92.2 (2.1)	<0.001	0.219	0.042	0.959	0.103	0.639
Vitamin B6 (µg) *(4.55µg/d)*	12.4 (0.2)	12.9 (0.2)	10.2 (0.2)	11.1 (0.3)	11.7 (0.4)	10.6 (0.2)	<0.001	<0.001	<0.001	<0.001	<0.001	<0.001
Folate (µg) *(1400µg/d)*	3091.5 (74.0)	3303.5 (72.7)	2415.2 (82.4)	2417.5 (89.6)	2352.9 (101.9)	2399.8 (86.5)	<0.001	<0.001	<0.001	<0.001	0.002	<0.001
Vitamin B12 (µg) *(8.4µg/d)*	4.3 (0.3)	2.9 (0.1)	4.1 (0.2)	3.2 (0.2)	3.3 (0.3)	3.5 (0.2)	<0.001	<0.001	<0.001	<0.001	<0.001	<0.001
Vitamin E (µg) *(33.5µg/d)*	39.8 (0.8)	42.1 (0.8)	29.5 (0.9)	33.7 (1.4)	36.7 (1.6)	27.8 (0.8)	<0.001	<0.001	<0.001	<0.001	<0.001	<0.001
Calcium(mg) *(4150mg/d)*	3756.5 (139.9)	3069.8 (71.0)	3303.2 (113.9)	2913.4 (116.9)	2934.7 (188.8)	3058.6 (102.8)	<0.001	<0.001	<0.001	<0.001	0.027	<0.001
Iron (mg) *(46.5mg/d)*	57.2 (1.4)	51.2 (1.0)	40.7 (1.4)	42.7 (1.6)	45.4 (2.4)	44.1 (1.4)	<0.001	<0.001	<0.001	0.208	0.855	0.308
Magnesium (mg) *(1250mg/d)*	2301.6 (44.2)	2186.3 (32.7)	1750.2 (43.0)	1824.9 (54.8)	2016.2 (78.9)	1794.0 (39.9)	<0.001	<0.001	<0.001	<0.001	<0.001	<0.001
Potassium (mg) *(12300mg/d)*	14514.1 (256.4)	14248.3 (229.6)	11970.7 (260.7)	12744.7 (359.7)	13850.2 (559.7)	11935.5 (249.6)	<0.001	<0.001	<0.001	<0.001	0.126	<0.001
Sodium (mg) *(8450mg/d)*	5011.5 (129.3)	4913.1 (105.1)	4234.9 (147.6)	3993.7 (151.4)	3742.5 (165.9)	4133.4 (150.8)	<0.001	<0.001	<0.001	<0.001	<0.001	<0.001
Zinc (mg) *(36.5mg/d)*	43.1 (0.9)	40.5 (0.6)	33.4 (0.8)	33.2 (1.0)	36.6 (1.4)	34.0 (0.8)	<0.001	<0.001	<0.001	<0.001	0.015	<0.001

[1] All data is mean (SEM).

[2] The recommended intake per household is calculated as the sum of the recommended intakes for each household member and is shown in brackets for each micronutrient. Household recommended intakes were calculated from the Australia New Zealand Nutrient Reference Values (33).

[3] Statistical comparison is between the current and healthier diets for low socioeconomic household (diet low SES); or current vs healthier diet for medium socioeconomic household (diet medium SES). For statistical significance, p<0.005.

SES, socioeconomic status.
