# Supplementary material for: Nutrient Dense, Low-Cost Foods Can Improve the Affordability and Quality of the New Zealand Diet—A Substitution Modeling Study
Source: Int J Environ Res Public Health. 2021 Jul 27;18(15):7950. doi: 10.3390/ijerph18157950 (PMC8345759; doi:10.3390/ijerph18157950)
Supplement: Supplementary file 1 [file ijerph-18-07950-s001.zip › Kellogg_Cereal Economics_SUPPLEMENTARY TABLE S5_IJERPH_NZ_20210709.pdf]

Supplementary Table S5. Individual component scores for the HEIFA-2013 (**A**) and DICE (**B**), for each ethnic group of both low and middle socioeconomic households.

A.

**HEIFA-2013 Scores[1]**	**Low socioeconomic household**			**Medium socioeconomic household**		
	**Current diet**	**Healthier diet**	***p*-value[2]**	**Current diet**	**Healthier diet**	***p*-value[2]**
**Māori**						
n	197	197		49	49	
Component 1: Discretionary	4.0 (0.3)	10.0 (0.0)	<0.001	5.0 (0.7)	10.0 (0.0)	<0.001
Component 2: Vegetables	1.9 (0.2)	3.2 (0.2)	<0.001	1.7 (0.4)	3.2 (0.4)	0.005
Component 3: Fruits	1.8 (0.2)	6.2 (0.3)	<0.001	3.2 (0.5)	7.2 (0.4)	<0.001
Component 4: Grains	2.3 (0.2)	5.2 (0.2)	<0.001	3.1 (0.4)	5.4 (0.5)	<0.001
Component 5: Meat	4.3 (0.3)	5.3 (0.3)	0.017	3.9 (0.6)	5.0 (0.6)	0.198
Component 6: Dairy	2.3 (0.2)	2.9 (0.2)	0.060	2.6 (0.4)	3.0 (0.5)	0.470
Component 7: Water	5.0 (0.0)	5.0 (0.0)	-	5.0 (0.0)	5.0 (0.0)	-
Component 8: Fat	1.8 (0.2)	8.3 (0.1)	<0.001	1.7 (0.3)	8.3 (0.3)	<0.001
Component 9: Sodium	6.3 (0.3)	10.0 (0.0)	<0.001	6.1 (0.6)	10.0 (0.0)	<0.001
Component 10: Added sugars	7.2 (0.3)	10.0 (0.0)	<0.001	7.0 (0.6)	10.0 (0.0)	<0.001
Component 11: Alcohol	5.0 (0.0)	5.0 (0.0)	-	5.0 (0.0)	5.0 (0.0)	-
HEIFA-2013 TOTAL SCORE	41.9 (0.8)	71.1 (0.8)	<0.001	44.2 (1.7)	72.1 (1.5)	<0.001
**Pacific**						
n	177	177		33	33	
Component 1: Discretionary	5.2 (0.3)	10.0 (0.0)	<0.001	4.6 (0.8)	10.0 (0.0)	<0.001
Component 2: Vegetables	2.8 (0.2)	3.5 (0.2)	0.011	2.7 (0.5)	3.4 (0.5)	0.330
Component 3: Fruits	2.0 (0.2)	5.9 (0.3)	<0.001	2.4 (0.5)	6.0 (0.7)	<0.001
Component 4: Grains	3.2 (0.2)	5.9 (0.3)	<0.001	3.1 (0.5)	5.4 (0.6)	0.0053
Component 5: Meat	5.5 (0.3)	6.0 (0.3)	0.274	4.5 (0.8)	5.6 (0.8)	0.348
Component 6: Dairy	2.1 (0.2)	2.8 (0.3)	0.046	2.8 (0.6)	3.2 (0.6)	0.675
Component 7: Water	5.0 (0.0)	5.0 (0.0)	-	5.0 (0.0)	5.0 (0.0)	-

Component 8: Fat	1.9 (0.2)	8.7 (0.1)	<0.001	1.6 (0.4)	8.4 (0.3)	<0.001
Component 9: Sodium	7.5 (0.3)	10.0 (0.0)	<0.001	6.6 (0.7)	10.0 (0.0)	<0.001
Component 10: Added sugars	7.1 (0.3)	10.0 (0.0)	<0.001	7.7 (0.7)	10.0 (0.0)	0.0014
Component 11: Alcohol	5.0 (0.0)	5.0 (0.0)	-	5.0 (0.0)	5.0 (0.0)	-
HEIFA-2013 TOTAL SCORE	47.4 (0.8)	72.8 (0.8)	<0.001	46.1 (1.8)	71.9 (2.0)	<0.001
**NZEO**						
n	91	91		120	120	
Component 1: Discretionary	4.8 (0.5)	10.0 (0.0)	<0.001	5.3 (0.4)	10.0 (0.0)	<0.001
Component 2: Vegetables	2.1 (0.2)	3.1 (0.3)	0.016	2.2 (0.2)	3.4 (0.2)	0.001
Component 3: Fruits	2.2 (0.3)	5.8 (0.4)	<0.001	3.1 (0.3)	6.4 (0.3)	<0.001
Component 4: Grains	2.7 (0.3)	5.5 (0.3)	<0.001	3.2 (0.3)	5.7 (0.3)	<0.001
Component 5: Meat	3.3 (0.4)	4.1 (0.4)	0.182	3.3 (0.4)	4.3 (0.4)	0.074
Component 6: Dairy	2.7 (0.3)	3.3 (0.4)	0.236	3.0 (0.3)	3.7 (0.3)	0.144
Component 7: Water	5.0 (0.0)	5.0 (0.0)	-	5.0 (0.0)	5.0 (0.0)	-
Component 8: Fat	1.8 (0.2)	7.7 (0.2)	<0.001	1.8 (0.2)	7.6 (0.2)	<0.001
Component 9: Sodium	7.3 (0.4)	10.0 (0.0)	<0.001	6.1 (0.4)	9.9 (0.0)	<0.001
Component 10: Added sugars	6.0 (0.5)	9.8 (0.1)	<0.001	7.1 (0.4)	10.0 (0.0)	<0.001
Component 11: Alcohol	5.0 (0.0)	5.0 (0.0)	-	5.0 (0.0)	5.0 (0.0)	-
HEIFA-2013 TOTAL SCORE	42.9 (1.2)	69.3 (1.1)	<0.001	45.3 (1.0)	71.0 (1.0)	<0.001

[1] All scores are mean (SEM).

[2] Statistical significance is set at p<0.05. A hyphen indicates that the values for comparison were exactly the same among all samples from within a group, and thus between both current vs healthier diets.

B.

DICE Scores[1]	Low socioeconomic household			Medium socioeconomic household		
	Current diet	Healthier diet	*p*-value[2]	Current diet	Healthier diet	*p*-value[2]
**Maori**						
n	194	194		67	67	
Component 1: Fruit	9.4 (0.3)	14.0 (0.2)	<0.001	10.0 (0.5)	13.7 (0.3)	<0.001
Component 2: Vegetables	4.1 (0.4)	8.1 (0.3)	<0.001	4.2 (0.6)	8.1 (0.6)	<0.001
Component 3: Breads and cereals	5.4 (0.3)	11.7 (0.3)	<0.001	4.8 (0.5)	10.5 (0.5)	<0.001
Component 4: Dairy and alternatives	2.9 (0.2)	3.5 (0.2)	0.055	2.9 (0.3)	3.2 (0.3)	0.613
Component 5: Protein foods	4.2 (0.3)	6.2 (0.3)	<0.001	3.7 (0.5)	5.2 (0.6)	0.061
Component 6: Number of meals	5.0 (0.0)	5.0 (0.0)	-	5.0 (0.0)	5.0 (0.0)	-
Component 7: Fat	1.0 (0.1)	4.8 (0.1)	<0.001	1.6 (0.3)	4.9 (0.1)	<0.001
Component 8: Salt	0.1 (0.1)	1.3 (0.2)	<0.001	0.3 (0.1)	1.4 (0.3)	<0.001
Component 9: Sugar	1.0 (0.1)	4.9 (0.1)	<0.001	0.9 (0.2)	5.0 (0.0)	<0.001
Component 10: Beverages	4.9 (0.5)	11.5 (0.3)	<0.001	6.0 (0.9)	11.6 (0.5)	<0.001
DICE TOTAL SCORE	37.9 (0.9)	70.9 (0.8)	<0.001	39.5 (1.8)	68.7 (1.4)	<0.001
**Pacific**						
n	243	243		33	33	
Component 1: Fruit	9.3 (0.3)	13.6 (0.2)	<0.001	10.6 (0.7)	14.0 (0.4)	<0.001
Component 2: Vegetables	4.2 (0.3)	8.4 (0.3)	<0.001	4.3 (0.9)	8.6 (0.8)	<0.001
Component 3: Breads and cereals	6.1 (0.3)	12.1 (0.3)	<0.001	5.3 (0.7)	11.4 (0.8)	<0.001
Component 4: Dairy and alternatives	1.9 (0.1)	2.5 (0.2)	0.0130	1.8 (0.4)	2.3 (0.5)	0.460
Component 5: Protein foods	5.1 (0.3)	6.5 (0.3)	<0.001	4.3 (0.8)	6.2 (0.8)	0.109
Component 6: Number of meals	5.0 (0.0)	5.0 (0.0)	-	5.0 (0.0)	5.0 (0.0)	-
Component 7: Fat	1.3 (0.1)	4.8 (0.1)	<0.001	1.2 (0.4)	4.8 (0.2)	<0.001
Component 8: Salt	0.3 (0.1)	1.6 (0.2)	<0.001	0.2 (0.2)	0.9 (0.3)	0.050
Component 9: Sugar	1.2 (0.1)	4.9 (0.0)	<0.001	2.1 (0.4)	5.0 (0.0)	<0.001
Component 10: Beverages	6.8 (0.5)	12.0 (0.2)	<0.001	5.9 (1.3)	12.5 (0.6)	<0.001

DICE TOTAL SCORE	41.3 (0.8)	71.3 (0.6)	<0.001	40.7 (2.6)	70.8 (1.9)	<0.001
**NZEO**						
n	42	42		70	70	
Component 1: Fruit	10.1 (0.6)	14.2 (0.3)	<0.001	10.1 (0.5)	13.6 (0.3)	<0.001
Component 2: Vegetables	4.7 (0.8)	8.3 (0.7)	<0.001	5.1 (0.6)	8.0 (0.5)	<0.001
Component 3: Breads and cereals	5.7 (0.7)	11.2 (0.7)	<0.001	5.6 (0.5)	10.6 (0.4)	<0.001
Component 4: Dairy and alternatives	3.4 (0.5)	4.6 (0.5)	0.112	3.0 (0.3)	3.2 (0.3)	0.686
Component 5: Protein foods	3.7 (0.7)	5.5 (0.7)	0.069	4.0 (0.6)	5.6 (0.6)	0.051
Component 6: Number of meals	5.0 (0.0)	5.0 (0.0)	-	5.0 (0.0)	5.0 (0.0)	-
Component 7: Fat	1.7 (0.4)	4.9 (0.1)	<0.001	1.5 (0.3)	4.9 (0.1)	<0.001
Component 8: Salt	0.2 (0.2)	1.4 (0.4)	0.003	0.1 (0.1)	1.6 (0.3)	<0.001
Component 9: Sugar	0.7 (0.3)	5.0 (0.0)	<0.001	1.1 (0.2)	5.0 (0.0)	<0.001
Component 10: Beverages	5.0 (1.1)	11.2 (0.6)	<0.001	4.5 (0.8)	11.0 (0.5)	<0.001
DICE TOTAL SCORE	40.3 (2.1)	71.3 (1.6)	<0.001	39.9 (1.7)	68.5 (1.3)	<0.001

[1] All scores are mean (SEM).

[2] Statistical significance is set at $p<0.05$. A hyphen indicates that the values for comparison were exactly the same among all samples from within a group, and thus between both current vs healthier diets.
